# Temporal Stability of the Salivary Microbiota in Oral Health

**DOI:** 10.1371/journal.pone.0147472

**Published:** 2016-01-22

**Authors:** Daniel Belstrøm, Palle Holmstrup, Allan Bardow, Alexis Kokaras, Nils-Erik Fiehn, Bruce J. Paster

**Affiliations:** 1 Section for Periodontology, Microbiology, and Community Dentistry, School of Dentistry, Faculty of Health and Medical Sciences, University of Copenhagen, Copenhagen, Denmark; 2 Department of Oral Medicine, School of Dentistry, University of Copenhagen, Copenhagen, Denmark; 3 The Forsyth Institute, Department of Microbiology, Cambridge, Massachusetts, United States of America; 4 Department of Immunology & Microbiology, University of Copenhagen, Copenhagen, Denmark; 5 Department of Oral Medicine, Infection & Immunity, Harvard School of Dental Medicine, Boston, Massachusetts, United States of America; University of Florida, UNITED STATES

## Abstract

**Objectives:**

Saliva is a biological fluid suitable for biomarker analysis, and differences in the salivary microbiota in oral health and disease have been reported. For such comparative analyses, time of sampling is critical since the bacterial composition may vary throughout the day, i.e., diurnal variation. The purpose of this study is to compare the salivary microbiome over time to determine the optimal time for sampling.

**Design:**

Stimulated saliva samples were collected from 5 orally healthy individuals in 4 h intervals for 24 h, and collection was repeated 7 days later (number of samples per person, n = 12, total number of samples, n = 60). Salivary microbiota was analyzed using the Human Oral Microbe Identification using Next Generation Sequencing (HOMI*NGS*), and statistical analysis was performed using the Kruskal-Wallis test with Benjamini-Hochberg’s correction for multiple comparisons, cluster analysis, principal component analysis and correspondence analysis.

**Results:**

From a total of 60 saliva samples, 477 probe targets were collectively identified with a mean number of probes per sample of 207 (range: 153–307). Little or no variation in microbial profiles within subjects was observed over time.

**Conclusions:**

Although there was considerable variation between subjects, microbial profiles within subjects were stable throughout a 24 hour period and after 1 week. Since there is little or no evidence of diurnal variation of the salivary microbiome, time of sampling of saliva is not critical for perturbation or other microbial studies.

## Introduction

Saliva-based screening for biomarkers associated with oral and general health status has gained considerable attention in the past decades [1–3], and as collection and analysis of blood samples and local oral microbial samples, including supra- and subgingival plaque samples, are often time-consuming procedures, testing of saliva is considered a simple, inexpensive and non-invasive alternative for biomarker analysis [4–5].

Interestingly, elevated salivary levels of pro-inflammatory cytokines including Tumor Necrosis Factor (TNF)-α [6–8] and Interleukin (IL)-1β [9–11] and molecules involved in tissue degradation such as Matrix Metalloproteinase (MMP)-8 and -9 [7,19,12–13] has been reported in saliva from patients with periodontitis. Furthermore, potential salivary biomarkers of various medical diseases, including Sjogren’s syndrome [14], breast cancer [15], pancreatic cancer [16] and Human Immunodeficiency Virus (HIV) [17] have been identified, suggesting that the biological composition of saliva may conceptually mirror the oral and general health status of the body [18]. However, the composition of the salivary proteome has been reported to express profound within-subject variability of salivary analytes. For example, a day-to-day variation of 67–201% of IL-1 β, IL-6, MMP-8, Prostaglandin E_2_ (PGE2), TNF- α, Interferon-α and Albumin was reported in unstimulated saliva samples collected from orally and systemically healthy adults during a two week period [19]. Consequently, it is crucial to determine within-subject variability of salivary analytes in healthy individuals before these measurements can be used for saliva-based screening of oral health and disease [11].

The composition of the salivary microbiota in oral health has been shown mostly comparable with that of the dorsum of the tongue, the throat and the tonsils, and is believed to constitute a mixture of bacteria shed from various oral surfaces [20]. Furthermore, the salivary microbiota has been addressed in cross-sectional studies using different molecular based methods, and has been reported to differentiate between individuals with periodontitis or dental caries as compared to individuals with oral health [21–23]. Therefore, salivary traceability of local microbial perturbations in relation to periodontitis and dental caries could potentially serve as a biomarker used in screening for oral diseases. However, before considering such a strategy further, it is pivotal to address possible influence of internal and external factors on the salivary bacterial composition. Previous studies have suggested that smoking [24] and other lifestyle-associated factors may have a general impact on the salivary microbiota [24–26]. Unfortunately, little is known about if the salivary microbiome varies in composition over a 24 hour period, e.g., diurnal variation, and previous studies have reported conflicting results in relation to long stability of the salivary microbiota [27–28]. However, such information is essential for oral microbiome studies to determine whether time of sampling would potentially influence variation in results.

Therefore, the purpose of this study was to learn whether the composition of the salivary microbiota shows diurnal variation. This was performed systematically by characterization of the salivary microbiota by using the Human Oral Microbe Identification using Next Generation Sequencing (HOMI*NGS*) technique on stimulated saliva samples collected from 5 orally healthy individuals at 12 different time-points.

## Material and Methods

### Study population

Five orally healthy, adult employees and students from the Department of Odontology, University of Copenhagen who fulfilled the inclusion criteria were included in this study. Inclusion criteria were age > 18 yrs. Exclusion criteria were as follows: self-reported presence of periodontitis or dental caries, current daily smoking, and treatment with local or systemic antibiotics within the past 3 months prior to participation. These criteria were established by questionnaire. Information of food-intake and oral hygiene procedures was recorded by the participant throughout the study period.

Thus, the study population was comprised of 5 individuals (3 employees and 2 graduate students), 4 males and 1 female with a mean age of 28.6 yrs. (range 24–36 yrs.) all being affiliated with the Department of Odontology, University of Copenhagen. Four out of 5 participants reported being systemically healthy with no daily consumption of medicine, whereas one participant was diagnosed with a mild form of Psoriasis.

All participants signed informed consent prior to participation, and the study was approved by The Ethics Committee for The Capital Region of Denmark (H-15000856) and reported to the Danish Data Authorization (2015-54-0970).

### Collection of saliva samples

Stimulated saliva samples were collected with 4 h intervals (12:00, 16:00, 20:00, 24:00, 04:00 [next day], and 08:00 [next day]) on 2 days with a 1 week interval. Collection of stimulated saliva was done by the participants themselves, as they were supplied with all materials necessary to perform collection of saliva. Thus, at the beginning of the study each participant was provided sterile packed plastic cups, transferring pipettes, plastic tubes, paraffin gums and a container with dry ice.

Collection of stimulated saliva samples was performed according to a previously described protocol [29]. In brief, the participants started by thorough flushing with a water rinse followed by chewing for 1 min with paraffin gum. Subsequently, participants were instructed to expel spit continuously for 1 min, and then to collect saliva in a plastic cup for an additional 3 min. Finally, saliva was transferred to a plastic tube and stored in a container with dry ice. After saliva had been collected for 1 day, all samples were brought to the laboratory and stored at -80 C until further analysis.

### Human Oral Microbe Identification using Next Generation Sequencing (HOMI*NGS*)

DNA isolation was performed as in previous reports of the salivary microbiota performed by our group, and according to the manufacturer’s guidelines (Roche, Mannheim, Germany), by specifications according to protocol Pathogen_Universal_200 [21–24]. The successor to the Human Oral Microbe Identification Microarray (HOMIM) is termed Human Oral Microbe Identification using Next Generation Sequencing (HOMI*NGS*) was utilized for bacterial identification [30]. At a glance, HOMI*NGS* is a newly developed molecular analysis, combining advances of considerably longer DNA-reads, generated through next generation sequencing (Illumina platform), and a subsequent BLAST of generated 16S rRNA reads against reference sequences of species-specific, custom designed 16S rDNA probes, enabling simultaneous identification of approximately 600 oral taxa at the species-level.

The laboratory procedures of HOMI*NGS* were performed by use of a modified protocol previously described [31]. Initially, 10–50 ng of DNA are PCR-amplified using V3-V4 forward (341F) AATGATACGGCGACCACCGAGATCTACACTATGGTAATTGTCCTACGGGAGGCAGCAG and reverse (806R) CAAGCAGAAGACGGCATACGAGATNNNNNNNNNNNNAGTCAGTCAGCCGGACTACHVGGGTWTCTAAT primers, followed by purification using AMPure beads. Subsequently, 100 ng of each library was pooled, gel-purified, and quantified using a qPCR. Finally, 12pM of the library mixture, spiked with 20% Phix, is sequenced by use of MiSeq (Illumina). In general, an average of >50,000 sequences of about 441 bp per sequence were obtained in each sample, and bad reads and chimeric sequences were removed from analyses. Subsequently, the sequenced 16S rRNA reads were blasted against species-specific, 16S rRNA-based oligonucleotide “probes”, many of which were originally designed for HOMIM, by use of a customized BLAST program (called ProbeSeq for HOMI*NGS*) developed at the Forsyth Institute, Cambridge, USA. Specifically, bacterial identification was therefore based on 598 oligonucleotide probes of 17 to 40 bases targeting individual oral bacterial species or, in some cases, a few closely-related species. In order to get nearly complete coverage, an additional panel of 94 genus-specific probes (a probe with a sequence that identifies a varied number of closely-related species within the same genus) was added to the analysis.

### Statistical analysis

Intra-individual differences (samples from the same individual) and differences between individuals at probe level were analyzed by Kruskal-Wallis test with Benjamini-Hochberg correction for multiple comparisons. Benjamini-Hochberg correction was used for control of false positive discoveries in positive dependent assumptions [32]. For this analysis an adjusted p-value < 0.01 was considered statistically significant. Analysis of comparability of microbial community profiles between samples was performed using cluster analysis, principal component analysis and correspondence analysis. GraphPad prism 5 (San Diego, California, USA) and MeV 4_8_1 [33] was used as statistical software.

## Results

### General findings

In 60 samples collected from 5 orally healthy individuals, positive identification for targets of 477 probe-sequences were observed (399 identifying a bacterial taxon and 78 identifying a bacterial genus) corresponding to a coverage of 62% of the 768 probe-sequences present in the probeseq database (a complete list of probes present in the Probeseq database and a list of bacterial probes identified are presented in S1 and S2 Files). The 5 most predominant genera identified were *Streptococcus*, *Haemophilus*, *Prevotella*, *Rothia* and *Neisseria* accounting for around 50% of targets identified. An average of 54,291 sequences (range 32,213–84,609) was generated out of which 44.7% (range 28.4%-60.3%) and 37.7% (range 20.2%-56.0%) were identified on species level and genus level respectively. In addition, an average of 17.6% (range 11.9%-33.8%) of the sequences generated could not be assigned to either a species-specific or a genus-specific probe-sequence based on BLAST against the HOMD database. (www.homd.org) [34].

### The salivary microbiota is host-specific

Based on comparison at probe level, a total of 181 and 287 probes were present with a significantly different frequency and different mean proportions respectively in samples from the 5 individuals (adjusted p-value < 0.01%). In addition, each of the 5 individuals had a personalized salivary bacterial fingerprint; thus, individual 1 was characterized by a combination of high levels of *Neisseria* Genus probe_2, *Haemophilus parainfluenzae* and *Haemophilus* genus probe_3. Individual 2 had a combination of high levels of *Gemella sanguinis*, *Prevotella melaninogenica*, *Veillonella* genus probe_2 and *Haemophilus parainfluenzae*. Individual 3 had substantial levels of *Neisseria subflava*. Individual 4 were characterized with high levels of *Neisseria* genus probe_2 and individual 5 expressed a combination of high levels of *Granulicatella elegans*, *Fusobacterium* genus probe_4, *Neisseria flavescens*, *Fusobacterium periodonticum* and *Gemella haemolysans*.

### Temporal stability of the salivary microbiota

**Fig 1:**
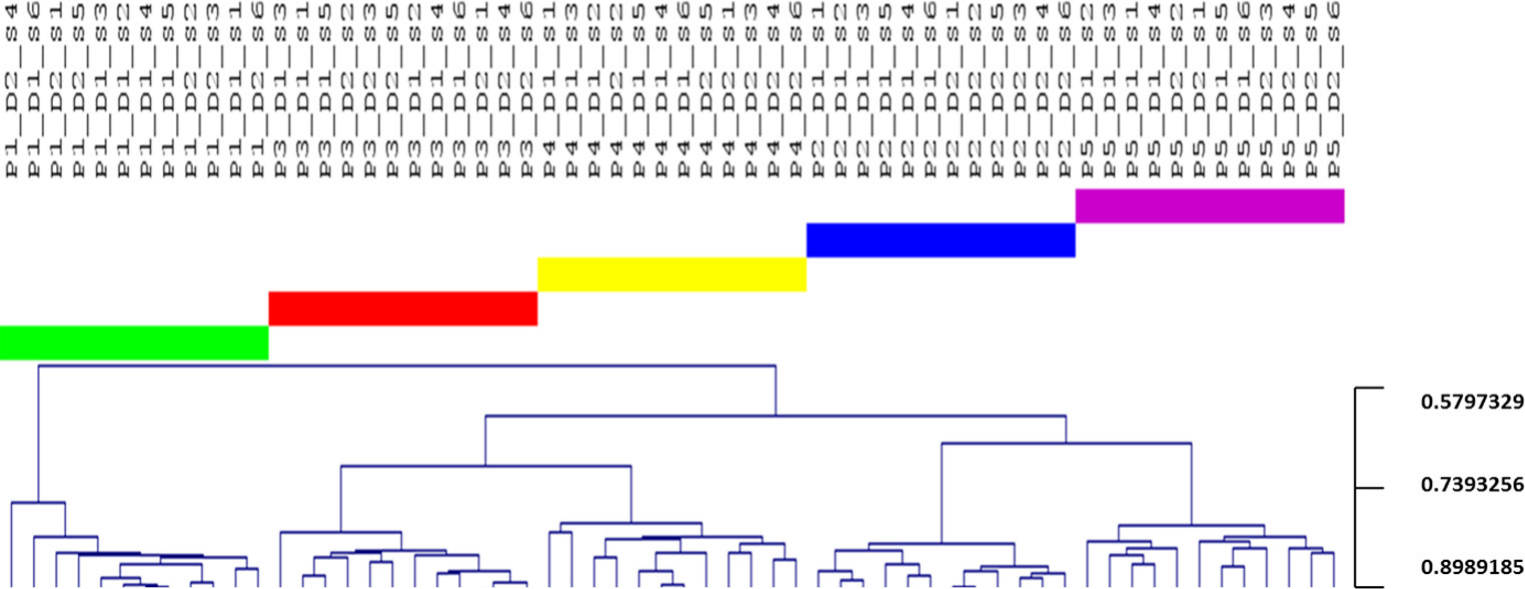
Cluster analysis. Cluster analysis based on Spearman Rank Correlation. Participant 1: green, Participant 2: blue, Participant 3: red, Participant 4: yellow, Participant 5: purple. Sample denotation: P1-P5 (Participant 1-Participant 5), D1-D2 (Day 1-Day 2), S1-S6 (Sample 1-Sample 6).

**Fig 2:**
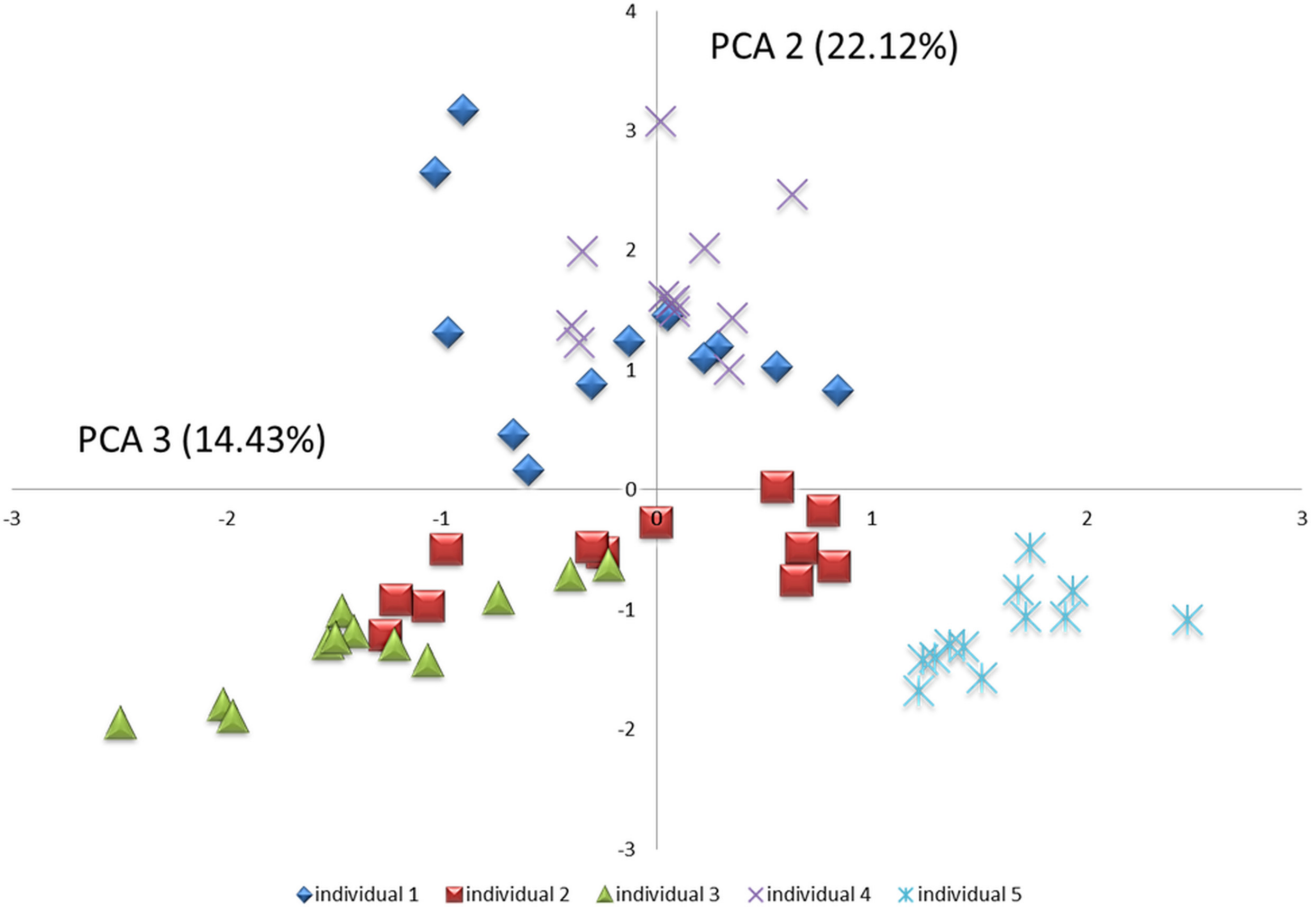
Principal component analysis. Principal component analysis visualized 2-dimensionally with axes expressed as the second and third most crucial components accounting for 36.6% of the variation of the dataset. Individual 1: blue square, individual 2: red square, individual 3: green triangle, individual 4: purple cross, individual 5: light blue cross.

**Fig 3:**
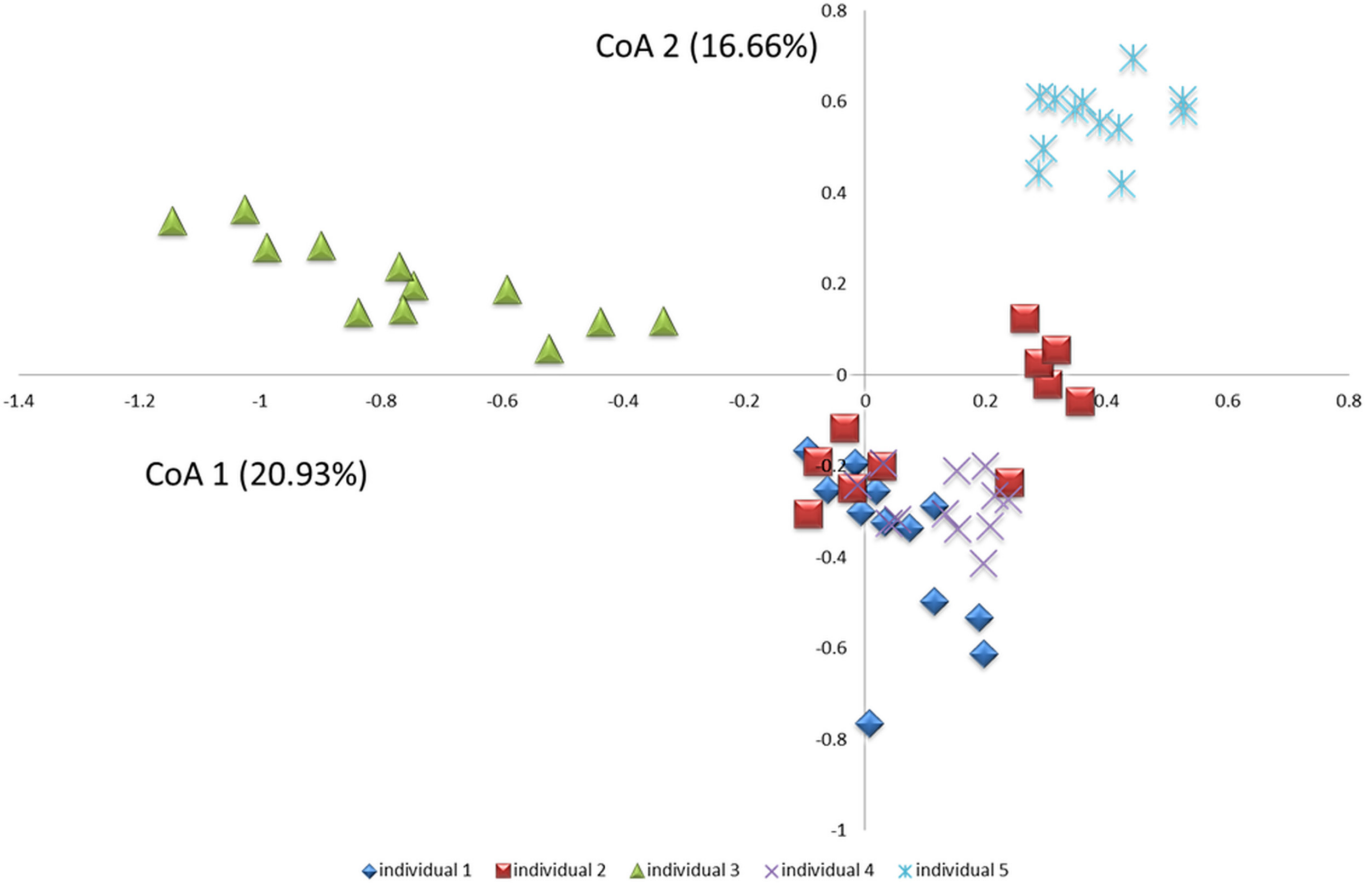
Correspondence analysis. Correspondence analysis visualized 2-dimensionally with axes expressed as the 2 most crucial inertia values accounting for a cumulative inertia of 37.6%. Individual 1: blue square, individual 2: red square, individual 3: green triangle, individual 4: purple cross, individual 5: light blue cross.

Comparison of samples from the 5 individuals by use of cluster analysis (Fig 1), principal component analysis (Fig 2) and correspondence analysis (Fig 3) demonstrated clear intra-individual clustering of samples with apparently no diurnal variation, as samples collected from various time-points within a 24 hour time-period clustered at random in each of the 5 individuals. In addition, random distribution of samples collected in week 1 and 2 were evident in 4 out of 5 individuals. In contrast, samples from individual 2 expressed separate clustering of samples collected in week 1 and week 2. This separate clustering was likely caused by a proportional increase of *Granulicatella* genus probe, *Leptotrichia*_sp_oral_taxon_417 and *Prevotella salivae* in combination with a proportional decrease of *Haemophilus parainfluenzae*, *Fusobacterium periodonticum*, *Neisseria flavescens*, *Porphyromonas*_sp_oral_taxon_279 and *Prevotella* genus probe_1 between samples collected in week 1 and week 2. Based on questionnaire-gathered information, it was revealed that individual 2 had switched toothpaste brand within the 2 weeks of sampling-a likely cause of the slight shift in microbial profiles.

## Discussion

The purpose of the present investigation was to address the degree to which the salivary microbiota in orally healthy individuals shows diurnal variation. This was performed by characterization of 60 stimulated saliva samples collected from 5 orally healthy individuals at 4 h intervals by use of the recently developed molecular method HOMI*NGS*. The main finding from this study was that stimulated saliva samples showed little or no variation in microbial profiles within subjects over time. To the best of our knowledge, this is the first study to systematically address short-term variations of the salivary microbiota.

In the present study, *Streptococcus* was the predominant genus associated with oral health, which is in accordance with previous studies carried out on saliva samples from orally healthy individuals using various molecular techniques [20, 24]. Notably, this finding demonstrates that comparable results on the salivary microbiota can be obtained when employing various molecular techniques. However, by use of HOMI*NGS*, we collectively identified targets of 477 different probes to be present in 60 saliva samples, which illustrate the more comprehensive nature of next generation sequencing-based molecular methods compared to microarrays when addressing microbial diversity of saliva samples [21–24]. Furthermore, in this study, putative periodontal pathogens as *Porphyromonas gingivalis* and *Aggregatibacter actinomycetemcomitans* were identified only in one and two out of 60 samples respectively, which is in accordance with HOMIM-based analysis of the salivary microbiota in oral health [24]. This observation reinforces the concept that these bacterial species in general are not typically detected in saliva samples from orally healthy individuals.

In the last decade, much attention has been given to saliva-based screening for biomarkers associated with oral health and disease [1–2], and increased salivary levels of analytes such as TNF- α, IL-1β and MMP-8 has been reported in patients with periodontitis when compared to orally healthy individuals [11,35]. In line, at group level different salivary bacterial community profiles have been shown between patients with periodontitis, dental caries and orally healthy individuals [21–24]. However, as it has been reported that proteomic profiles of saliva show considerable levels of within-subject variability [19], it is considered paramount to determine within-subject variability of potential proteomic biomarkers in healthy individuals as a prerequisite for protein-based diagnostic testing of saliva [11]. Essentially, this may compromise the feasibility for utilizing saliva-based screening of proteomic profiles performed routinely at the dental clinic.

Therefore, based on data from protein-based analysis of saliva, we considered it crucial to address the issue if the composition of the salivary microbiota also showed diurnal variation. Also, since previous studies on the salivary microbiota in oral health and disease performed by our group were carried out using samples collected at various time-points, it was essential to learn if data from these studies was potentially biased by diurnal variation [21–24]. Interestingly, in previous reports, the composition of the salivary microbiota has been suggested to remain stable for 5 days [28] and up to 7 yrs. [27]. To determine the potential diurnal variation of the salivary microbiome, we used a systematic collection of stimulated saliva samples in 4 h intervals, and repeated this approach with a one week interval, to ensure that data obtained provided comprehensive information on short-term alterations of the salivary microbiota in orally healthy individuals. Based on data from this study, the composition of the salivary microbiota showed largely no diurnal variation. Notably, this result confirms previous findings suggesting that oral health and disease associates with different salivary bacterial profiles regardless the time of sampling [21–24]. From a practical point of view, this is essential for the potential to utilize bacterial profiles of saliva as stabile biomarkers for screening of oral health and disease.

In conclusion, data from this study are a proof of concept conceptually underlining that diurnal variation does not affect the salivary microbiome. Consequently, the data suggest that time of sampling to determine the salivary microbiome does not have to be considered to obtain valid results. Future studies are needed to reveal if other oral habitats, including the sulcus, buccal epithelia, and tongue dorsum, are affected by diurnal variation.

## Supporting Information

S1 File List of all probes present in the Probeseq database. Complete list of all probes present in the Probeseq database listed alphabetically with taxon-specific probes first followed by genus probes.
(DOCX)

S2 File List of probes identified. Complete list of the 477 probes identified (399 recognizing a bacterial taxon and 78 recognizing a bacterial genus). Probes are listed according to their proportional presence (%) across samples in decreasing order.
(DOCX)
