# Supplementary material for: Temporal Stability of the Salivary Microbiota in Oral Health
Source: PLoS One. 2016 Jan 22;11(1):e0147472. doi: 10.1371/journal.pone.0147472 (PMC4723053; doi:10.1371/journal.pone.0147472)
Supplement: S1 File — Complete list of all probes present in the Probeseq database listed alphabetically with taxon-specific probes first followed by genus probes. (DOCX) [file pone.0147472.s001.docx]

| **Probe ID** | **HOT** | **Organism Name** |
| --- | --- | --- |
| **AB-01** | **389** | *Abiotrophia_defectiva* |
| **AC-01** | **554** | *Acinetobacter_baumannii* |
| **AC-02** | **408** | *Acinetobacter_sp_oral_taxon_408* |
| **AC-03** | **183** | *Actinobaculum_sp_oral_taxon_183* |
| **AC-04** | **850** | *Actinomyces_cardiffensis* |
| **AC-06** | **617** | *Actinomyces_georgiae* |
| **AC-07** | **618** | *Actinomyces_gerencseriae* |
| **AC-08** | **866** | *Actinomyces_graevenitzii* |
| **AC-09** | **645** | *Actinomyces_israelii* |
| **AC-10** | **849** | *Actinomyces_johnsonii* |
| **AC-11** | **852** | *Actinomyces_massiliensis* |
| **AC-12** | **671** | *Actinomyces_meyeri* |
| **AC-13** | **176** | *Actinomyces_naeslundii* |
| **AC-14** | **701** | *Actinomyces_odontolyticus* |
| **AC-15** | **708** | *Actinomyces_oricola* |
| **AC-16** | **746** | *Actinomyces_radicidentis* |
| **AC-17** | **169** | *Actinomyces*_sp_oral_taxon_169 |
| **AC-18** | **170** | *Actinomyces*_sp_oral_taxon_170 |
| **AC-19** | **171** | *Actinomyces*_sp_oral_taxon_171 |
| **AC-20** | **172** | *Actinomyces*_sp_oral_taxon_172 |
| **AC-21** | **175** | *Actinomyces*_sp_oral_taxon_175 |
| **AC-22** | **178** | *Actinomyces*_sp_oral_taxon_178 |
| **AC-23** | **180** | *Actinomyces*_sp_oral_taxon_180 |
| **AC-24** | **181** | *Actinomyces*_sp_oral_taxon_181 |
| **AC-25** | **414** | *Actinomyces*_sp_oral_taxon_414 |
| **AC-26** | **446** | *Actinomyces*_sp_oral_taxon_446 |
| **AC-27** | **448** | *Actinomyces*_sp_oral_taxon_448 |
| **AC-28** | **525** | *Actinomyces*_sp_oral_taxon_525 |
| **AC-29** | **848** | *Actinomyces*_sp_oral_taxon_848 |
| **AC-30** | **877** | *Actinomyces*_sp_oral_taxon_877 |
| **AC-31** | **896** | *Actinomyces*_sp_oral_taxon_896 |
| **AC-32** | **897** | *Actinomyces*_sp_oral_taxon_897 |
| **AC-33** | **179** | *Actinomyces_timonensis* |
| **AC-34** | **688** | *Actinomyces_viscosus* |
| **AC-35** | **866** | *Actinomyces_graevenitzii* |
| **AC-36** | **176** | *Actinomyces_naeslundii* |
| **AC-37** | **701** | *Actinomyces_odontolyticus* |
| **AC-38** | **701** | *Actinomyces_odontolyticus* |
| **AC-39** | **746** | *Actinomyces_radicidentis* |
| **AC-40** | **525** | *Actinomyces*_sp_oral_taxon_525 |
| **AC-41** | **179** | *Actinomyces_timonensis* |
| **AG-01** | **531** | *Aggregatibacter_actinomycetemcomitans* |
| **AG-03** | **720** | *Aggregatibacter_paraphrophilus* |
| **AG-05** | **512** | *Aggregatibacter*_sp_oral_taxon_512 |
| **AG-06** | **513** | *Aggregatibacter*_sp_oral_taxon_513 |
| **AG-07** | **485** | *Agrobacterium_tumefaciens* |
| **AG-08** | **458** | *Aggregatibacter*_sp_oral_taxon_458 |
| **AG-09** | **720** | *Aggregatibacter_paraphrophilus* |
| **AL-01** | **831** | *Alloiococcus_otitis* |
| **AL-02** | **302** | *Alloprevotella_rava* |
| **AL-03** | **308** | *Alloprevotella*_sp_oral_taxon_308 |
| **AL-04** | **473** | *Alloprevotella*_sp_oral_taxon_473 |
| **AL-05** | **474** | *Alloprevotella*_sp_oral_taxon_474 |
| **AL-06** | **912** | *Alloprevotella*_sp_oral_taxon_912 |
| **AL-07** | **913** | *Alloprevotella*_sp_oral_taxon_913 |
| **AL-08** | **914** | *Alloprevotella*_sp_oral_taxon_914 |
| **AL-09** | **466** | *Alloprevotella_tannerae* |
| **AL-10** | **198** | *Alloscardovia_omnicolens* |
| **AL-11** | **914** | *Alloprevotella*_sp_oral_taxon_914 |
| **AN-01** | **859** | *Anaerococcus_lactolyticus* |
| **AN-03** | **121** | *Anaeroglobus_geminatus* |
| **AQ-01** | **660** | *Aquamicrobium_lusatiense* |
| **AR-01** | **811** | *Arcanobacterium_haemolyticum* |
| **AR-02** | **190** | *Arsenicicoccus*_sp_oral_taxon_190 |
| **AT-01** | **674** | *Atopobium_minutum* |
| **AT-02** | **723** | *Atopobium_parvulum* |
| **AT-03** | **750** | *Atopobium_rimae* |
| **AT-04** | **199** | *Atopobium*_sp_oral_taxon_199 |
| **AT-05** | **416** | *Atopobium*_sp_oral_taxon_416 |
| **AT-06** | **810** | *Atopobium*_sp_oral_taxon_810 |
| **AT-07** | **814** | *Atopobium_vaginae* |
| **AT-08** | **723** | *Atopobium_parvulum* |
| **AT-09** | **750** | *Atopobium_rimae* |
| **BA-01** | **45** | *Bacillus_clausii* |
| **BA-02** | **272** | *Bacteroidaceae*[G-1]_sp_oral_taxon_272 |
| **BA-03** | **274** | *Bacteroidales*[G-2]_sp_oral_taxon_274 |
| **BA-04** | **911** | *Bacteroidales*[G-3]_sp_oral_taxon_911 |
| **BA-05** | **630** | *Bacteroides_heparinolyticus* |
| **BA-06** | **787** | *Bacteroides_tectus* |
| **BA-07** | **465** | *Bacteroides_zoogleoformans* |
| **BA-08** | **280** | *Bacteroidetes*[G-3]_sp_oral_taxon_280 |
| **BA-09** | **281** | *Bacteroidetes*[G-3]_sp_oral_taxon_281 |
| **BA-10** | **365** | *Bacteroidetes*[G-3]_sp_oral_taxon_365 |
| **BA-11** | **436** | *Bacteroidetes*[G-3]_sp_oral_taxon_436 |
| **BA-12** | **503** | *Bacteroidetes*[G-3]_sp_oral_taxon_503 |
| **BA-13** | **899** | *Bacteroidetes*[G-3]_sp_oral_taxon_899 |
| **BA-14** | **509** | *Bacteroidetes*[G-4]_sp_oral_taxon_509 |
| **BA-15** | **505** | *Bacteroidetes*[G-5]_sp_oral_taxon_505 |
| **BA-16** | **507** | *Bacteroidetes*[G-5]_sp_oral_taxon_507 |
| **BA-17** | **511** | *Bacteroidetes*[G-5]_sp_oral_taxon_511 |
| **BA-18** | **516** | *Bacteroidetes*[G-6]_sp_oral_taxon_516 |
| **BA-19** | **511** | *Bacteroidetes*[G-5]_sp_oral_taxon_511 |
| **BD-01** | **39** | *Bdellovibrio*_sp_oral_taxon_039 |
| **BE-01** | **319** | *Bergeyella*_sp_oral_taxon_319 |
| **BE-02** | **322** | *Bergeyella*_sp_oral_taxon_322 |
| **BE-03** | **900** | *Bergeyella*_sp_oral_taxon_900 |
| **BE-04** | **907** | *Bergeyella*_sp_oral_taxon_907 |
| **BI-01** | **407** | *Bifidobacteriaceae*[G-2]_sp_oral_taxon_407 |
| **BI-02** | **895** | *Bifidobacterium*_animalis_subsp_animalis |
| **BI-03** | **895** | *Bifidobacterium*_animalis_subsp_lactis |
| **BI-04** | **889** | *Bifidobacterium_breve* |
| **BI-05** | **588** | *Bifidobacterium_dentium* |
| **BI-06** | **862** | *Bifidobacterium_longum* |
| **BI-07** | **891** | *Bifidobacterium_scardovii* |
| **BR-01** | **590** | *Brevundimonas_diminuta* |
| **BU-01** | **603** | *Bulleidia_extructa* |
| **BU-02** | **94** | *Butyrivibrio*_sp_oral_taxon_094 |
| **CA-01** | **575** | *Campylobacter_concisus* |
| **CA-02** | **575** | *Campylobacter_concisus* |
| **CA-03** | **580** | *Campylobacter_curvus* |
| **CA-04** | **623** | *Campylobacter_gracilis* |
| **CA-06** | **44** | *Campylobacter*_sp_oral_taxon_044 |
| **CA-07** | **776** | *Campylobacter_sputorum* |
| **CA-08** | **842** | *Campylobacter_ureolyticus* |
| **CA-09** | **337** | *Capnocytophaga_gingivalis* |
| **CA-10** | **325** | *Capnocytophaga_granulosa* |
| **CA-11** | **627** | *Capnocytophaga_haemolytica* |
| **CA-12** | **329** | *Capnocytophaga_leadbetteri* |
| **CA-13** | **700** | *Capnocytophaga_ochracea* |
| **CA-14** | **323** | *Capnocytophaga*_sp_oral_taxon_323 |
| **CA-15** | **324** | *Capnocytophaga*_sp_oral_taxon_324 |
| **CA-17** | **332** | *Capnocytophaga*_sp_oral_taxon_332 |
| **CA-18** | **334** | *Capnocytophaga*_sp_oral_taxon_334 |
| **CA-19** | **335** | *Capnocytophaga*_sp_oral_taxon_335 |
| **CA-20** | **336** | *Capnocytophaga*_sp_oral_taxon_336 |
| **CA-21** | **338** | *Capnocytophaga*_sp_oral_taxon_338 |
| **CA-22** | **380** | *Capnocytophaga*_sp_oral_taxon_380 |
| **CA-23** | **412** | *Capnocytophaga*_sp_oral_taxon_412 |
| **CA-24** | **863** | *Capnocytophaga*_sp_oral_taxon_863 |
| **CA-25** | **864** | *Capnocytophaga*_sp_oral_taxon_864 |
| **CA-26** | **878** | *Capnocytophaga*_sp_oral_taxon_878 |
| **CA-27** | **901** | *Capnocytophaga*_sp_oral_taxon_901 |
| **CA-28** | **902** | *Capnocytophaga*_sp_oral_taxon_902 |
| **CA-29** | **903** | *Capnocytophaga*_sp_oral_taxon_903 |
| **CA-30** | **775** | *Capnocytophaga_sputigena* |
| **CA-31** | **633** | *Cardiobacterium_hominis* |
| **CA-32** | **540** | *Cardiobacterium_valvarum* |
| **CA-34** | **451** | *Catonella*_sp_oral_taxon_451 |
| **CA-35** | **2** | *Caulobacter*_sp_oral_taxon_002 |
| **CE-01** | **726** | *Centipeda_periodontii* |
| **CH-01** | **733** | *Chlamydophila_pneumoniae* |
| **CH-02** | **439** | *Chloroflexi*[G-1]_sp_oral_taxon_439 |
| **CH-03** | **439** | *Chloroflexi*[G-1]_sp_oral_taxon_439 |
| **CL-01** | **93** | *Clostridiales*[F-1][G-1]_sp_oral_taxon_093 |
| **CL-02** | **402** | *Clostridiales*[F-1][G-2]_sp_oral_taxon_402 |
| **CL-03** | **75** | *Clostridiales*[F-2][G-1]_sp_oral_taxon_075 |
| **CL-04** | **85** | *Clostridiales*[F-2][G-2]_sp_oral_taxon_085 |
| **CL-05** | **366** | *Clostridiales*[F-2][G-3]_sp_oral_taxon_366 |
| **CL-06** | **381** | *Clostridiales*[F-2][G-3]_sp_oral_taxon_381 |
| **CL-07** | **876** | *Clostridiales*[F-3][G-1]_sp_oral_taxon_876 |
| **CL-08** | **75** | *Clostridiales*[F-2][G-1]_sp_oral_taxon_075 |
| **CO-01** | **591** | *Corynebacterium_diphtheriae* |
| **CO-02** | **595** | *Corynebacterium_durum* |
| **CO-03** | **666** | *Corynebacterium_matruchotii* |
| **CO-04** | **835** | *Corynebacterium_mucifaciens* |
| **CO-05** | **184** | *Corynebacterium*_sp_oral_taxon_184 |
| **CO-06** | **853** | *Corynebacterium_urealyticum* |
| **CO-07** | **591** | *Corynebacterium_diphtheriae* |
| **CO-08** | **591** | *Corynebacterium_diphtheriae* |
| **CR-01** | **579** | *Cryptobacterium_curtum* |
| **DE-01** | **23** | *Delftia_acidovorans* |
| **DE-02** | **41** | *Desulfobulbus*_sp_oral_taxon_041 |
| **DE-03** | **703** | *Desulfomicrobium_orale* |
| **DE-04** | **605** | *Desulfovibrio_fairfieldensis* |
| **DE-05** | **40** | *Desulfovibrio*_sp_oral_taxon_040 |
| **DI-01** | **118** | *Dialister_invisus* |
| **DI-02** | **843** | *Dialister_micraerophilus* |
| **DI-03** | **736** | *Dialister_pneumosintes* |
| **DI-04** | **119** | *Dialister*_sp_oral_taxon_119 |
| **DI-05** | **502** | *Dialister*_sp_oral_taxon_502 |
| **DI-06** | **368** | *Dietzia*_sp_oral_taxon_368 |
| **DI-07** | **502** | *Dialister*_sp_oral_taxon_502 |
| **DI-08** | **502** | *Dialister*_sp_oral_taxon_502 |
| **DI-09** | **502** | *Dialister*_sp_oral_taxon_502 |
| **DO-01** | **813** | *Dolosigranulum_pigrum* |
| **EG-01** | **654** | *Eggerthella_lenta* |
| **EG-02** | **569** | *Eggerthia_catenaformis* |
| **EI-01** | **577** | *Eikenella_corrodens* |
| **EI-02** | **11** | *Eikenella*_sp_oral_taxon_011 |
| **EN-02** | **604** | *Enterococcus_faecalis* |
| **ER-01** | **904** | *Erysipelothrichaceae*[G-1]_sp_oral_taxon_904 |
| **ER-02** | **905** | *Erysipelothrichaceae*[G-1]_sp_oral_taxon_905 |
| **ER-03** | **484** | *Erysipelothrix_tonsillarum* |
| **ER-04** | **747** | *Erythromicrobium_ramosum* |
| **EU-01** | **655** | *Eubacterium_limosum* |
| **EU-02** | **105** | *Eubacterium*[11][G-1]_infirmum |
| **EU-03** | **467** | *Eubacterium*[11][G-1]_sulci |
| **EU-04** | **557** | *Eubacterium*[11][G-3]_brachy |
| **EU-05** | **759** | *Eubacterium*[11][G-5]_saphenum |
| **EU-06** | **673** | *Eubacterium*[11][G-6]_minutum |
| **EU-07** | **694** | *Eubacterium*[11][G-6]_nodatum |
| **EU-08** | **377** | *Eubacterium*[11][G-7]_yurii |
| **EU-09** | **694** | *Eubacterium*[11][G-6]_nodatum |
| **EU-10** | **377** | *Eubacterium*[11][G-7]_yurii |
| **EU-11** | **655** | *Eubacterium_limosum* |
| **FI-01** | **539** | *Filifactor_alocis* |
| **FI-02** | **662** | *Finegoldia_magna* |
| **FL-01** | **318** | *Flavobacteriales*[G-1]_sp_oral_taxon_318 |
| **FL-02** | **321** | *Flavobacteriales*[G-1]_sp_oral_taxon_321 |
| **FL-03** | **320** | *Flavobacteriales*[G-2]_sp_oral_taxon_320 |
| **FR-01** | **363** | *Fretibacterium_fastidiosum* |
| **FR-04** | **360** | *Fretibacterium*_sp_oral_taxon_360 |
| **FR-05** | **361** | *Fretibacterium*_sp_oral_taxon_361 |
| **FR-06** | **362** | *Fretibacterium*_sp_oral_taxon_362 |
| **FU-01** | **860** | *Fusobacterium_gonidiaformans* |
| **FU-02** | **860** | *Fusobacterium_gonidiaformans* |
| **FU-04** | **690** | *Fusobacterium_necrophorum* |
| **FU-05** | **420** | *Fusobacterium_nucleatum*_subsp_animalis |
| **FU-06** | **420** | *Fusobacterium_nucleatum*_subsp_animalis |
| **FU-07** | **698** | *Fusobacterium_nucleatum*_subsp_nucleatum |
| **FU-08** | **202** | *Fusobacterium_nucleatum*_subsp_polymorphum |
| **FU-09** | **200** | *Fusobacterium_nucleatum*_subsp_vincentii |
| **FU-10** | **201** | *Fusobacterium_periodonticum* |
| **FU-11** | **205** | *Fusobacterium*_sp_oral_taxon_205 |
| **GA-01** | **829** | *Gardnerella_vaginalis* |
| **GE-01** | **555** | *Gemella_bergeri* |
| **GE-02** | **626** | *Gemella_haemolysans* |
| **GE-03** | **46** | *Gemella_morbillorum* |
| **GE-04** | **757** | *Gemella_sanguinis* |
| **GE-05** | **46** | *Gemella_morbillorum* |
| **GN-01** | **871** | GN02[G-1]_sp_oral_taxon_871 |
| **GN-02** | **872** | GN02[G-1]_sp_oral_taxon_872 |
| **GN-03** | **873** | GN02[G-2]_sp_oral_taxon_873 |
| **GR-02** | **596** | *Granulicatella_elegans* |
| **HA-02** | **821** | *Haemophilus_ducreyi* |
| **HA-04** | ***** | *Haemophilus_parahaemolyticus* |
| **HA-05** | **718** | *Haemophilus_parainfluenzae* |
| **HA-06** | **35** | *Haemophilus*_sp_oral_taxon_035 |
| **HE-01** | **812** | *Helicobacter_pylori* |
| **JO-01** | **635** | *Johnsonella_ignava* |
| **JO-02** | **166** | *Johnsonella*_sp_oral_taxon_166 |
| **JO-03** | **777** | *Jonquetella_anthropi* |
| **KI-02** | **646** | *Kingella_kingae* |
| **KI-03** | **706** | *Kingella_oralis* |
| **KI-04** | **459** | *Kingella*_sp_oral_taxon_459 |
| **LA-01** | **82** | *Lachnoanaerobaculum_orale* |
| **LA-02** | **494** | *Lachnoanaerobaculum_saburreum* |
| **LA-03** | **83** | *Lachnoanaerobaculum*_sp_oral_taxon_083 |
| **LA-04** | **89** | *Lachnoanaerobaculum*_sp_oral_taxon_089 |
| **LA-05** | **496** | *Lachnoanaerobaculum*_sp_oral_taxon_496 |
| **LA-06** | **107** | *Lachnoanaerobaculum_umeaense* |
| **LA-07** | **88** | *Lachnospiraceae*[G-2]_sp_oral_taxon_088 |
| **LA-08** | **96** | Lachnospiraceae[G-2]_sp_oral_taxon_096 |
| **LA-09** | **100** | *Lachnospiraceae*[G-3]_sp_oral_taxon_100 |
| **LA-10** | **80** | *Lachnospiraceae*[G-5]_sp_oral_taxon_080 |
| **LA-11** | **455** | *Lachnospiraceae*[G-5]_sp_oral_taxon_455 |
| **LA-12** | **90** | *Lachnospiraceae*[G-6]_sp_oral_taxon_090 |
| **LA-13** | **86** | *Lachnospiraceae*[G-7]_sp_oral_taxon_086 |
| **LA-14** | **163** | *Lachnospiraceae*[G-7]_sp_oral_taxon_163 |
| **LA-15** | **500** | *Lachnospiraceae*[G-8]_sp_oral_taxon_500 |
| **LA-16** | **558** | *Lactobacillus_brevis* |
| **LA-17** | **816** | *Lactobacillus_coleohominis* |
| **LA-18** | **608** | *Lactobacillus_fermentum* |
| **LA-20** | **838** | *Lactobacillus_iners* |
| **LA-21** | **839** | *Lactobacillus_jensenii* |
| **LA-22** | **424** | *Lactobacillus_kisonensis* |
| **LA-23** | **418** | *Lactobacillus_parafarraginis* |
| **LA-24** | **818** | *Lactobacillus_reuteri* |
| **LA-25** | **756** | *Lactobacillus_salivarius* |
| **LA-26** | **52** | *Lactobacillus_sp_oral_taxon_052* |
| **LA-27** | **51** | *Lactobacillus_vaginalis* |
| **LA-28** | **804** | *Lactococcus_lactis* |
| **LA-29** | **22** | *Lautropia_mirabilis* |
| **LA-30** | **88** | *Lachnospiraceae*[G-2]_sp_oral_taxon_088 |
| **LA-31** | **100** | *Lachnospiraceae*[G-3]_sp_oral_taxon_100 |
| **LA-32** | **100** | *Lachnospiraceae*[G-3]_sp_oral_taxon_100 |
| **LE-01** | **24** | *Leptothrix*_sp_oral_taxon_024 |
| **LE-02** | **25** | *Leptothrix*_sp_oral_taxon_025 |
| **LE-04** | **845** | *Leptotrichia_goodfellowii* |
| **LE-06** | **213** | *Leptotrichia_hongkongensis* |
| **LE-07** | **214** | *Leptotrichia_shahii* |
| **LE-08** | **212** | *Leptotrichia*_sp_oral_taxon_212 |
| **LE-09** | **215** | *Leptotrichia*_sp_oral_taxon_215 |
| **LE-10** | **217** | *Leptotrichia*_sp_oral_taxon_217 |
| **LE-11** | **218** | *Leptotrichia*_sp_oral_taxon_218 |
| **LE-12** | **219** | *Leptotrichia*_sp_oral_taxon_219 |
| **LE-13** | **221** | *Leptotrichia*_sp_oral_taxon_221 |
| **LE-14** | **223** | *Leptotrichia*_sp_oral_taxon_223 |
| **LE-15** | **392** | *Leptotrichia*_sp_oral_taxon_392 |
| **LE-16** | **417** | *Leptotrichia*_sp_oral_taxon_417 |
| **LE-17** | **462** | *Leptotrichia*_sp_oral_taxon_462 |
| **LE-18** | **463** | *Leptotrichia*_sp_oral_taxon_463 |
| **LE-19** | **498** | *Leptotrichia*_sp_oral_taxon_498 |
| **LE-20** | **847** | *Leptotrichia*_sp_oral_taxon_847 |
| **LE-21** | **879** | *Leptotrichia*_sp_oral_taxon_879 |
| **LE-22** | **222** | *Leptotrichia_wadei* |
| **LE-23** | **210** | *Leptotrichiaceae*[G-1]_sp_oral_taxon_210 |
| **LE-24** | **220** | *Leptotrichiaceae*[G-1]_sp_oral_taxon_220 |
| **LE-25** | **223** | *Leptotrichia*_sp_oral_taxon_223 |
| **LE-26** | **215** | *Leptotrichia*_sp_oral_taxon_215 |
| **LY-01** | **614** | *Lysinibacillus_fusiformis* |
| **ME-01** | **122** | *Megasphaera_micronuciformis* |
| **ME-02** | **123** | *Megasphaera*_sp_oral_taxon_123 |
| **ME-03** | **123** | *Megasphaera*_sp_oral_taxon_123 |
| **ME-04** | **841** | *Megasphaera*_sp_oral_taxon_841 |
| **MI-01** | **185** | *Microbacterium*_sp_oral_taxon_185 |
| **MI-02** | **684** | *Mitsuokella_multacida* |
| **MI-03** | **131** | *Mitsuokella*_sp_oral_taxon_131 |
| **MI-04** | **521** | *Mitsuokella*_sp_oral_taxon_521 |
| **MI-05** | **521** | *Mitsuokella*_sp_oral_taxon_521 |
| **MI-06** | **521** | *Mitsuokella*_sp_oral_taxon_521 |
| **MO-01** | **830** | *Mobiluncus_mulieris* |
| **MO-02** | **593** | *Mogibacterium_diversum* |
| **MO-03** | **742** | *Mogibacterium_pumilum* |
| **MO-04** | **42** | *Mogibacterium_timidum* |
| **MO-05** | **504** | *Mollicutes*[G-1]_sp_oral_taxon_504 |
| **MO-06** | **906** | *Mollicutes*[G-2]_sp_oral_taxon_906 |
| **MY-01** | **823** | *Mycobacterium_leprae* |
| **MY-02** | **561** | *Mycoplasma_buccale* |
| **MY-03** | **606** | *Mycoplasma_faucium* |
| **MY-04** | **607** | *Mycoplasma_fermentans* |
| **MY-05** | **616** | *Mycoplasma_genitalium* |
| **MY-06** | **632** | *Mycoplasma_hominis* |
| **MY-07** | **656** | *Mycoplasma_lipophilum* |
| **MY-08** | **704** | *Mycoplasma_orale* |
| **MY-09** | **732** | *Mycoplasma_pneumoniae* |
| **MY-10** | **754** | *Mycoplasma_salivarium* |
| **NE-01** | **13** | *Neisseria_bacilliformis* |
| **NE-02** | **598** | *Neisseria_elongata* |
| **NE-03** | **610** | *Neisseria_flavescens* |
| **NE-04** | **621** | *Neisseria_gonorrhoeae* |
| **NE-05** | **649** | *Neisseria_lactamica* |
| **NE-06** | **669** | *Neisseria_meningitidis* |
| **NE-08** | **729** | *Neisseria_pharyngis* |
| **NE-09** | **764** | *Neisseria_sicca* |
| **NE-10** | **18** | *Neisseria*_sp_oral_taxon_018 |
| **NE-11** | **20** | *Neisseria*_sp_oral_taxon_020 |
| **NE-12** | **499** | *Neisseria*_sp_oral_taxon_499 |
| **NE-13** | **523** | *Neisseria*_sp_oral_taxon_523 |
| **NE-14** | **92** | *Neisseria_weaveri* |
| **NE-15** | **729** | *Neisseria_pharyngis* |
| **NE-16** | **610** | *Neisseria_flavescens* |
| **NE-17** | **476** | *Neisseria_subflava* |
| **OL-01** | **806** | *Olsenella_profusa* |
| **OL-02** | **807** | *Olsenella*_sp_oral_taxon_807 |
| **OL-03** | **809** | *Olsenella*_sp_oral_taxon_809 |
| **OL-04** | **38** | *Olsenella_uli* |
| **OR-01** | **457** | *Oribacterium_sinus* |
| **OR-03** | **102** | *Oribacterium*_sp_oral_taxon_102 |
| **OR-04** | **108** | *Oribacterium*_sp_oral_taxon_108 |
| **OR-05** | **108** | Oribacterium_sp_oral_taxon_108 |
| **OR-06** | **108** | *Oribacterium*_sp_oral_taxon_108 |
| **OT-01** | **894** | *Ottowia*_sp_oral_taxon_894 |
| **OT-02** | **894** | *Ottowia*_sp_oral_taxon_894 |
| **PA-01** | **786** | *Paenibacillus*_sp_oral_taxon_786 |
| **PA-02** | **586** | *Parascardovia_denticolens* |
| **PA-03** | **111** | *Parvimonas_micra* |
| **PA-04** | **110** | *Parvimonas*_sp_oral_taxon_110 |
| **PE-01** | **167** | *Peptococcus*_sp_oral_taxon_167 |
| **PE-02** | **168** | *Peptococcus*_sp_oral_taxon_168 |
| **PE-03** | **548** | *Peptoniphilus_asaccharolyticus* |
| **PE-04** | **840** | *Peptoniphilus_indolicus* |
| **PE-05** | **648** | *Peptoniphilus_lacrimalis* |
| **PE-06** | **375** | *Peptoniphilus*_sp_oral_taxon_375 |
| **PE-07** | **386** | *Peptoniphilus*_sp_oral_taxon_386 |
| **PE-08** | **836** | *Peptoniphilus*_sp_oral_taxon_836 |
| **PE-09** | **383** | *Peptostreptococcaceae*[11][G-1]_sp_oral_taxon_383 |
| **PE-10** | **91** | *Peptostreptococcaceae*[11][G-2]_sp_oral_taxon_091 |
| **PE-11** | **382** | *Peptostreptococcaceae*[11][G-3]_sp_oral_taxon_382 |
| **PE-12** | **495** | *Peptostreptococcaceae*[11][G-3]_sp_oral_taxon_495 |
| **PE-13** | **103** | *Peptostreptococcaceae*[11][G-4]_sp_oral_taxon_103 |
| **PE-14** | **369** | *Peptostreptococcaceae*[11][G-4]_sp_oral_taxon_369 |
| **PE-15** | **493** | *Peptostreptococcaceae*[11][G-5]_sp_oral_taxon_493 |
| **PE-16** | **81** | *Peptostreptococcaceae*[11][G-7]_sp_oral_taxon_081 |
| **PE-17** | **106** | *Peptostreptococcaceae*[11][G-7]_sp_oral_taxon_106 |
| **PE-18** | **113** | *Peptostreptococcaceae*[13][G-1]_sp_oral_taxon_113 |
| **PE-19** | **790** | *Peptostreptococcaceae*[13][G-2]_sp_oral_taxon_790 |
| **PE-20** | **542** | *Peptostreptococcus_anaerobius* |
| **PE-21** | **112** | *Peptostreptococcus_stomatis* |
| **PE-22** | **167** | *Peptococcus*_sp_oral_taxon_167 |
| **PE-23** | **91** | *Peptostreptococcaceae*[11][G-2]_sp_oral_taxon_091 |
| **PE-24** | **91** | *Peptostreptococcaceae*[11][G-2]_sp_oral_taxon_091 |
| **PO-01** | **547** | *Porphyromonas_asaccharolytica* |
| **PO-02** | **283** | *Porphyromonas_catoniae* |
| **PO-03** | **273** | *Porphyromonas_endodontalis* |
| **PO-05** | **619** | *Porphyromonas_gingivalis* |
| **PO-06** | **275** | *Porphyromonas*_sp_oral_taxon_275 |
| **PO-09** | **279** | *Porphyromonas*_sp_oral_taxon_279 |
| **PO-10** | **284** | *Porphyromonas*_sp_oral_taxon_284 |
| **PO-11** | **285** | *Porphyromonas*_sp_oral_taxon_285 |
| **PO-12** | **395** | *Porphyromonas*_sp_oral_taxon_395 |
| **PO-13** | **785** | *Porphyromonas_uenonis* |
| **PO-14** | **283** | *Porphyromonas_catoniae* |
| **PO-15** | **283** | *Porphyromonas_catoniae* |
| **PO-16** | **283** | *Porphyromonas_catoniae* |
| **PO-17** | **283** | *Porphyromonas_catoniae* |
| **PO-18** | **283** | *Porphyromonas_catoniae* |
| **PO-19** | **275** | *Porphyromonas*_sp_oral_taxon_275 |
| **PO-20** | **275** | *Porphyromonas*_sp_oral_taxon_275 |
| **PO-21** | **277** | *Porphyromonas*_sp_oral_taxon_277 |
| **PO-22** | **277** | *Porphyromonas*_sp_oral_taxon_277 |
| **PO-23** | **278** | *Porphyromonas*_sp_oral_taxon_278 |
| **PO-24** | **279** | *Porphyromonas*_sp_oral_taxon_279 |
| **PO-25** | **279** | *Porphyromonas*_sp_oral_taxon_279 |
| **PR-01** | **553** | *Prevotella_baroniae* |
| **PR-02** | **556** | *Prevotella_bivia* |
| **PR-03** | **560** | *Prevotella_buccae* |
| **PR-04** | **562** | *Prevotella_buccalis* |
| **PR-05** | **583** | *Prevotella_dentalis* |
| **PR-06** | **291** | *Prevotella_denticola* |
| **PR-07** | **600** | *Prevotella_enoeca* |
| **PR-08** | **782** | *Prevotella_fusca* |
| **PR-09** | **298** | *Prevotella_histicola* |
| **PR-10** | **643** | *Prevotella_intermedia* |
| **PR-11** | **658** | *Prevotella_loescheii* |
| **PR-12** | **289** | *Prevotella_maculosa* |
| **PR-13** | **665** | *Prevotella_marshii* |
| **PR-14** | **469** | *Prevotella_melaninogenica* |
| **PR-15** | **378** | *Prevotella_micans* |
| **PR-16** | **685** | *Prevotella_multiformis* |
| **PR-17** | **794** | *Prevotella_multisaccharivorax* |
| **PR-18** | **693** | *Prevotella_nigrescens* |
| **PR-19** | **705** | *Prevotella_oralis* |
| **PR-20** | **311** | *Prevotella_oris* |
| **PR-21** | **288** | *Prevotella_oulorum* |
| **PR-22** | **714** | *Prevotella_pallens* |
| **PR-23** | **303** | *Prevotella_pleuritidis* |
| **PR-24** | **781** | *Prevotella_saccharolytica* |
| **PR-25** | **307** | *Prevotella_salivae* |
| **PR-26** | **885** | *Prevotella_scopos* |
| **PR-27** | **795** | *Prevotella_shahii* |
| **PR-28** | **292** | *Prevotella*_sp_oral_taxon_292 |
| **PR-29** | **292** | *Prevotella*_sp_oral_taxon_292 |
| **PR-30** | **293** | *Prevotella*_sp_oral_taxon_293 |
| **PR-31** | **296** | *Prevotella*_sp_oral_taxon_296 |
| **PR-33** | **300** | *Prevotella*_sp_oral_taxon_300 |
| **PR-34** | **300** | *Prevotella*_sp_oral_taxon_300 |
| **PR-35** | **301** | *Prevotella*_sp_oral_taxon_301 |
| **PR-36** | **304** | *Prevotella*_sp_oral_taxon_304 |
| **PR-37** | **305** | *Prevotella*_sp_oral_taxon_305 |
| **PR-38** | **306** | *Prevotella*_sp_oral_taxon_306 |
| **PR-39** | **309** | *Prevotella*_sp_oral_taxon_309 |
| **PR-40** | **310** | *Prevotella*_sp_oral_taxon_310 |
| **PR-41** | **315** | *Prevotella*_sp_oral_taxon_315 |
| **PR-42** | **317** | *Prevotella*_sp_oral_taxon_317 |
| **PR-43** | **376** | *Prevotella*_sp_oral_taxon_376 |
| **PR-44** | **396** | *Prevotella*_sp_oral_taxon_396 |
| **PR-45** | **443** | *Prevotella*_sp_oral_taxon_443 |
| **PR-46** | **472** | *Prevotella*_sp_oral_taxon_472 |
| **PR-47** | **475** | *Prevotella*_sp_oral_taxon_475 |
| **PR-48** | **515** | *Prevotella*_sp_oral_taxon_515 |
| **PR-49** | **526** | *Prevotella*_sp_oral_taxon_526 |
| **PR-50** | **820** | *Prevotella*_sp_oral_taxon_820 |
| **PR-51** | **572** | *Prevotella_veroralis* |
| **PR-52** | **191** | *Propionibacterium_acidifaciens* |
| **PR-53** | **530** | *Propionibacterium_acnes* |
| **PR-54** | **552** | *Propionibacterium_avidum* |
| **PR-55** | **739** | *Propionibacterium_propionicum* |
| **PR-56** | **192** | *Propionibacterium*_sp_oral_taxon_192 |
| **PR-57** | **193** | *Propionibacterium*_sp_oral_taxon_193 |
| **PR-58** | **194** | *Propionibacterium*_sp_oral_taxon_194 |
| **PR-59** | **915** | *Propionibacterium*_sp_oral_taxon_915 |
| **PR-60** | **676** | *Proteus*_mirabilis |
| **PR-61** | **600** | *Prevotella*_enoeca |
| **PR-62** | **782** | *Prevotella_fusca* |
| **PR-63** | **782** | *Prevotella_fusca* |
| **PR-64** | **643** | *Prevotella_intermedia* |
| **PR-65** | **643** | *Prevotella_intermedia* |
| **PR-66** | **658** | *Prevotella_loescheii* |
| **PR-67** | **658** | *Prevotella_loescheii* |
| **PR-68** | **665** | *Prevotella_marshii* |
| **PR-69** | **378** | *Prevotella_micans* |
| **PR-70** | **714** | *Prevotella_pallens* |
| **PR-71** | **781** | *Prevotella_saccharolytica* |
| **PR-72** | **781** | *Prevotella_saccharolytica* |
| **PR-73** | **317** | *Prevotella*_sp_oral_taxon_317 |
| **PR-74** | **317** | *Prevotella*_sp_oral_taxon_317 |
| **PR-75** | **552** | *Propionibacterium_avidum* |
| **PS-01** | **536** | *Pseudomonas_aeruginosa* |
| **PS-02** | **612** | *Pseudomonas_fluorescens* |
| **PS-03** | **834** | *Pseudomonas_otitidis* |
| **PS-04** | **32** | *Pseudomonas*_sp_oral_taxon_032 |
| **PS-06** | **538** | *Pseudoramibacter_alactolyticus* |
| **PY-01** | **357** | *Pyramidobacter_piscolens* |
| **RH-01** | **28** | *Rhodocyclus*_sp_oral_taxon_028 |
| **RO-01** | **188** | *Rothia_aeria* |
| **RO-02** | **587** | *Rothia_dentocariosa* |
| **RO-03** | **681** | *Rothia_mucilaginosa* |
| **SC-01** | **642** | *Scardovia_inopinata* |
| **SC-02** | **195** | *Scardovia_wiggsiae* |
| **SE-01** | **124** | *Selenomonas_artemidis* |
| **SE-02** | **139** | *Selenomonas_dianae* |
| **SE-03** | **125** | *Selenomonas_flueggei* |
| **SE-04** | **130** | *Selenomonas_noxia* |
| **SE-05** | **130** | *Selenomonas_noxia* |
| **SE-06** | **133** | *Selenomonas*_sp_oral_taxon_133 |
| **SE-07** | **134** | *Selenomonas*_sp_oral_taxon_134 |
| **SE-08** | **136** | *Selenomonas*_sp_oral_taxon_136 |
| **SE-09** | **137** | *Selenomonas*_sp_oral_taxon_137 |
| **SE-10** | **138** | *Selenomonas*_sp_oral_taxon_138 |
| **SE-11** | **143** | *Selenomonas*_sp_oral_taxon_143 |
| **SE-12** | **146** | *Selenomonas*_sp_oral_taxon_146 |
| **SE-13** | **149** | *Selenomonas*_sp_oral_taxon_149 |
| **SE-14** | **388** | *Selenomonas*_sp_oral_taxon_388 |
| **SE-15** | **442** | *Selenomonas*_sp_oral_taxon_442 |
| **SE-16** | **478** | *Selenomonas*_sp_oral_taxon_478 |
| **SE-17** | **501** | *Selenomonas*_sp_oral_taxon_501 |
| **SE-18** | **151** | *Selenomonas_sputigena* |
| **SE-19** | **151** | *Selenomonas_sputigena* |
| **SE-21** | **124** | *Selenomonas_artemidis* |
| **SE-22** | **139** | *Selenomonas_dianae* |
| **SE-24** | **133** | *Selenomonas*_sp_oral_taxon_133 |
| **SE-25** | **134** | *Selenomonas*_sp_oral_taxon_134 |
| **SE-26** | **134** | *Selenomonas*_sp_oral_taxon_134 |
| **SE-27** | **136** | *Selenomonas*_sp_oral_taxon_136 |
| **SE-28** | **137** | *Selenomonas*_sp_oral_taxon_137 |
| **SE-29** | **137** | *Selenomonas*_sp_oral_taxon_137 |
| **SE-30** | **478** | *Selenomonas*_sp_oral_taxon_478 |
| **SE-31** | **151** | *Selenomonas_sputigena* |
| **SE-32** | **151** | *Selenomonas_sputigena* |
| **SE-33** | **151** | *Selenomonas_sputigena* |
| **SH-01** | **95** | *Shuttleworthia_satelles* |
| **SI-01** | **683** | *Simonsiella_muelleri* |
| **SL-01** | **602** | *Slackia_exigua* |
| **SN-01** | **844** | *Sneathia_amnionii* |
| **SN-02** | **837** | *Sneathia_sanguinegens* |
| **SO-01** | **678** | *Solobacterium_moorei* |
| **SR-01** | **345** | SR1[G-1]_sp_oral_taxon_345 |
| **SR-02** | **874** | SR1[G-1]_sp_oral_taxon_874 |
| **SR-03** | **875** | SR1[G-1]_sp_oral_taxon_875 |
| **ST-03** | **663** | *Stenotrophomonas_maltophilia* |
| **ST-04** | **419** | *Stomatobaculum_longum* |
| **ST-05** | **97** | *Stomatobaculum*_sp_oral_taxon_097 |
| **ST-06** | **373** | *Stomatobaculum*_sp_oral_taxon_373 |
| **ST-07** | **910** | *Stomatobaculum*_sp_oral_taxon_910 |
| **ST-08** | **537** | *Streptococcus_agalactiae* |
| **ST-09** | **543** | *Streptococcus_anginosus* |
| **ST-10** | **576** | *Streptococcus_constellatus* |
| **ST-11** | **578** | *Streptococcus_cristatus* |
| **ST-12** | **594** | *Streptococcus_downei* |
| **ST-14** | **644** | *Streptococcus_intermedius* |
| **ST-15** | **686** | *Streptococcus_mutans* |
| **ST-16** | **411** | *Streptococcus_parasanguinis_II* |
| **ST-18** | **745** | *Streptococcus_pyogenes* |
| **ST-20** | **758** | *Streptococcus_sanguinis* |
| **ST-21** | **768** | *Streptococcus_sobrinus* |
| **ST-22** | **64** | *Streptococcus*_sp_oral_taxon_064 |
| **ST-23** | **66** | *Streptococcus*_sp_oral_taxon_066 |
| **ST-24** | **68** | *Streptococcus*_sp_oral_taxon_068 |
| **ST-25** | **69** | *Streptococcus*_sp_oral_taxon_069 |
| **ST-26** | **431** | *Streptococcus*_sp_oral_taxon_431 |
| **ST-27** | **486** | *Streptococcus*_sp_oral_taxon_486 |
| **ST-28** | **487** | *Streptococcus*_sp_oral_taxon_487 |
| **ST-29** | **663** | *Stenotrophomonas_maltophilia* |
| **SY-01** | **435** | *Syntrophomonadaceae*[8][G-1]_sp_oral_taxon_435 |
| **TA-01** | **613** | *Tannerella_forsythia* |
| **TA-02** | **286** | *Tannerella*_sp_oral_taxon_286 |
| **TA-03** | **808** | *Tannerella*_sp_oral_taxon_808 |
| **TA-04** | **916** | *Tannerella*_sp_oral_taxon_916 |
| **TA-05** | **916** | *Tannerella*_sp_oral_taxon_916 |
| **TA-06** | **916** | *Tannerella*_sp_oral_taxon_916 |
| **TA-07** | **916** | *Tannerella*_sp_oral_taxon_916 |
| **TM-01** | **346** | TM7[G-1]_sp_oral_taxon_346 |
| **TM-02** | **347** | TM7[G-1]_sp_oral_taxon_347 |
| **TM-03** | **348** | TM7[G-1]_sp_oral_taxon_348 |
| **TM-04** | **349** | TM7[G-1]_sp_oral_taxon_349 |
| **TM-05** | **352** | TM7[G-1]_sp_oral_taxon_352 |
| **TM-06** | **353** | TM7[G-1]_sp_oral_taxon_353 |
| **TM-07** | **488** | TM7[G-1]_sp_oral_taxon_488 |
| **TM-08** | **350** | TM7[G-2]_sp_oral_taxon_350 |
| **TM-09** | **351** | TM7[G-3]_sp_oral_taxon_351 |
| **TM-10** | **355** | TM7[G-4]_sp_oral_taxon_355 |
| **TM-11** | **356** | TM7[G-5]_sp_oral_taxon_356 |
| **TM-12** | **437** | TM7[G-5]_sp_oral_taxon_437 |
| **TM-13** | **348** | TM7[G-1]_sp_oral_taxon_348 |
| **TR-01** | **541** | *Treponema_amylovorum* |
| **TR-02** | **584** | *Treponema_denticola* |
| **TR-03** | **584** | *Treponema_denticola* |
| **TR-04** | **653** | *Treponema_lecithinolyticum* |
| **TR-05** | **664** | *Treponema_maltophilum* |
| **TR-06** | **805** | *Treponema_pallidum* |
| **TR-07** | **724** | *Treponema_parvum* |
| **TR-08** | **725** | *Treponema_pectinovorum* |
| **TR-09** | **743** | *Treponema_putidum* |
| **TR-10** | **769** | *Treponema_socranskii* |
| **TR-11** | **769** | *Treponema_socranskii* |
| **TR-12** | **226** | *Treponema*_sp_oral_taxon_226 |
| **TR-13** | **227** | *Treponema*_sp_oral_taxon_227 |
| **TR-14** | **228** | *Treponema*_sp_oral_taxon_228 |
| **TR-15** | **230** | *Treponema*_sp_oral_taxon_230 |
| **TR-17** | **232** | *Treponema*_sp_oral_taxon_232 |
| **TR-18** | **234** | *Treponema*_sp_oral_taxon_234 |
| **TR-19** | **235** | *Treponema*_sp_oral_taxon_235 |
| **TR-20** | **236** | *Treponema*_sp_oral_taxon_236 |
| **TR-21** | **238** | *Treponema*_sp_oral_taxon_238 |
| **TR-22** | **239** | *Treponema*_sp_oral_taxon_239 |
| **TR-23** | **242** | *Treponema*_sp_oral_taxon_242 |
| **TR-24** | **246** | *Treponema*_sp_oral_taxon_246 |
| **TR-25** | **247** | *Treponema*_sp_oral_taxon_247 |
| **TR-26** | **249** | *Treponema*_sp_oral_taxon_249 |
| **TR-27** | **250** | *Treponema*_sp_oral_taxon_250 |
| **TR-28** | **252** | *Treponema*_sp_oral_taxon_252 |
| **TR-29** | **253** | *Treponema*_sp_oral_taxon_253 |
| **TR-30** | **254** | *Treponema*_sp_oral_taxon_254 |
| **TR-31** | **255** | *Treponema*_sp_oral_taxon_255 |
| **TR-32** | **256** | *Treponema*_sp_oral_taxon_256 |
| **TR-33** | **257** | *Treponema*_sp_oral_taxon_257 |
| **TR-34** | **258** | *Treponema*_sp_oral_taxon_258 |
| **TR-35** | **260** | *Treponema*_sp_oral_taxon_260 |
| **TR-36** | **262** | *Treponema*_sp_oral_taxon_262 |
| **TR-37** | **263** | *Treponema*_sp_oral_taxon_263 |
| **TR-38** | **264** | *Treponema*_sp_oral_taxon_264 |
| **TR-39** | **265** | *Treponema*_sp_oral_taxon_265 |
| **TR-40** | **268** | *Treponema*_sp_oral_taxon_268 |
| **TR-41** | **268** | *Treponema*_sp_oral_taxon_268 |
| **TR-42** | **269** | *Treponema*_sp_oral_taxon_269 |
| **TR-43** | **270** | *Treponema*_sp_oral_taxon_270 |
| **TR-44** | **271** | *Treponema_*sp_oral_taxon_271 |
| **TR-45** | **490** | *Treponema*_sp_oral_taxon_490 |
| **TR-46** | **508** | *Treponema_*sp_oral_taxon_508 |
| **TR-47** | **517** | *Treponema*_sp_oral_taxon_517 |
| **TR-48** | **518** | *Treponema*_sp_oral_taxon_518 |
| **TR-49** | **29** | *Treponema_vincentii* |
| **TR-50** | **769** | *Treponema_socranskii* |
| **TR-51** | **769** | *Treponema_socranskii* |
| **TR-52** | **228** | *Treponema*_sp_oral_taxon_228 |
| **TR-53** | **262** | *Treponema*_sp_oral_taxon_262 |
| **TR-54** | **508** | *Treponema*_sp_oral_taxon_508 |
| **TU-01** | **832** | *Turicella_otitidis* |
| **VA-01** | **717** | *Variovorax_paradoxus* |
| **VE-02** | **887** | *Veillonella_denticariosi* |
| **VE-03** | **160** | *Veillonella_dispar* |
| **VE-05** | **161** | *Veillonella_parvula* |
| **VE-06** | **161** | *Veillonella_parvula* |
| **VE-07** | **158** | *Veillonella_rogosae* |
| **VE-08** | **780** | *Veillonella*_sp_oral_taxon_780 |
| **VE-09** | **917** | *Veillonella*_sp_oral_taxon_917 |
| **VE-10** | **129** | *Veillonellaceae*[G-1]_sp_oral_taxon_129 |
| **VE-12** | **135** | *Veillonellaceae*[G-1]_sp_oral_taxon_135 |
| **VE-13** | **145** | *Veillonellaceae*[G-1]_sp_oral_taxon_145 |
| **VE-14** | **148** | *Veillonellaceae*[G-1]_sp_oral_taxon_148 |
| **VE-15** | **155** | *Veillonellaceae*[G-1]_sp_oral_taxon_155 |
| **VE-16** | **483** | *Veillonellaceae*[G-1]_sp_oral_taxon_483 |
| **VE-17** | **918** | *Veillonellacea*e[G-1]_sp_oral_taxon_918 |
| **VE-18** | **887** | *Veillonella_denticariosi* |
| **VE-19** | **155** | *Veillonellaceae*[G-1]_sp_oral_taxon_155 |
| **VE-20** | **524** | *Veillonella_atypica* |
| **VE-21** | **524** | *Veillonella_atypica* |
| **GP-001** |  | *Achromobacter*_Genus_probe |
| **GP-002** |  | *Acinetobacter*_Genus_probe |
| **GP-097** |  | *Actinomyces*_Genus_probe_1 |
| **GP-098** |  | *Actinomyces*_Genus_probe_2 |
| **GP-003** |  | *Actinomyces*_Genus_probe_3 |
| **GP-004** |  | *Actinomyces*_Genus_probe_4 |
| **GP-099** |  | *Aggregatibacter*_Genus_probe_1 |
| **GP-100** |  | *Aggregatibacter*_Genus_probe_2 |
| **GP-005** |  | *Alloprevotella*_Genus_probe |
| **GP-101** |  | *Anaerococcus*_Genus_probe_1 |
| **GP-006** |  | *Anaerococcus*_Genus_probe_2 |
| **GP-007** |  | *Aquamicrobium*_Genus_probe |
| **GP-008** |  | *Arcanobacterium*_Genus_probe |
| **GP-009** |  | *Atopobium*_Genus_probe |
| **GP-010** |  | *Bacteroides*_Genus_probe |
| **GP-011** |  | *Bacteroidetes*[G-3]_Genus_probe |
| **GP-012** |  | *Bacteroidetes*[G-5]_Genus_probe |
| **GP-013** |  | *Bartonella*_Genus_probe |
| **GP-014** |  | *Bifidobacterium*_Genus_probe_1 |
| **GP-015** |  | *Bifidobacterium*_Genus_probe_2 |
| **GP-016** |  | *Bordetella*_Genus_probe |
| **GP-017** |  | *Brevundimonas*_Genus_probe |
| **GP-018** |  | *Burkholderia*_Genus_probe |
| **GP-102** |  | *Campylobacter*_Genus_probe_1 |
| **GP-019** |  | *Campylobacter*_Genus_probe_2 |
| **GP-103** |  | *Capnocytophaga*_Genus_probe_1 |
| **GP-020** |  | *Capnocytophaga*_Genus_probe_2 |
| **GP-021** |  | *Capnocytophaga*_Genus_probe_3 |
| **GP-022** |  | *Cardiobacterium*_Genus_probe |
| **GP-023** |  | *Catonella*_Genus_probe |
| **GP-024** |  | *Chlamydophila*_Genus_probe |
| **GP-025** |  | *Corynebacterium*_Genus_probe |
| **GP-026** |  | *Desulfobulbus*_Genus_probe |
| **GP-093** |  | *Dialister*_Genus_probe_1 |
| **GP-094** |  | *Dialister*_Genus_probe_2 |
| **GP-028** |  | *Dietzia*_Genus_probe |
| **GP-029** |  | *Eggerthella*_Genus_probe |
| **GP-105** |  | *Enterococcus*_Genus_probe_1 |
| **GP-106** |  | *Enterococcus*_Genus_probe_2 |
| **GP-030** |  | *Enterococcus*_Genus_probe_3 |
| **GP-031** |  | *Erysipelothrichaceae*_Genus_probe |
| **GP-032** |  | *Erysipelothrix*_Genus_probe |
| **GP-033** |  | *Escherichia*_Genus_probe |
| **GP-034** |  | *Eubacterium*_Genus_probe_1 |
| **GP-035** |  | *Eubacterium*_Genus_probe_2 |
| **GP-036** |  | *Filifactor*_Genus_probe |
| **GP-107** |  | *Fretibacterium*_Genus_probe_1 |
| **GP-108** |  | *Fretibacterium*_Genus_probe_2 |
| **GP-037** |  | *Fretibacterium*_Genus_probe_3 |
| **GP-109** |  | *Fusobacterium*_Genus_probe_1 |
| **GP-095** |  | *Fusobacterium*_Genus_probe_2 |
| **GP-096** |  | *Fusobacterium*_Genus_probe_3 |
| **GP-038** |  | *Fusobacterium*_Genus_probe_4 |
| **GP-039** |  | *Gemella*_Genus_probe |
| **GP-110** |  | *Granulicatella*_Genus_probe |
| **GP-111** |  | *Haemophilus*_Genus_probe_1 |
| **GP-112** |  | *Haemophilus*_Genus_probe_2 |
| **GP-040** |  | *Haemophilus*_Genus_probe_3 |
| **GP-041** |  | *Helicobacter*_Genus_probe |
| **GP-042** |  | *Johnsonella*_Genus_probe |
| **GP-113** |  | *Kingella*_Genus_probe_1 |
| **GP-114** |  | *Kingella*_Genus_probe_2 |
| **GP-043** |  | *Kytococcus*_Genus_probe |
| **GP-044** |  | *Lachnoanaerobaculum*_Genus_probe |
| **GP-115** |  | *Lactobacillus*_Genus_probe_1 |
| **GP-045** |  | *Lactobacillus*_Genus_probe_2 |
| **GP-046** |  | *Lactobacillus*_Genus_probe_3 |
| **GP-047** |  | *Lactobacillus*_Genus_probe_4 |
| **GP-048** |  | *Lactobacillus*_Genus_probe_5 |
| **GP-116** |  | *Leptotrichia*_Genus_probe_1 |
| **GP-117** |  | *Leptotrichia*_Genus_probe_2 |
| **GP-049** |  | *Leptotrichia*_Genus_probe_3 |
| **GP-050** |  | *Leptotrichia*_Genus_probe_4 |
| **GP-051** |  | *Leptotrichiaceae*_Genus_probe |
| **GP-052** |  | *Listeria*_Genus_probe |
| **GP-053** |  | *Lysinibacillus*_Genus_probe |
| **GP-054** |  | *Mitsuokella_*Genus_probe |
| **GP-055** |  | *Mobiluncus*_Genus_probe |
| **GP-056** |  | *Mogibacterium*_Genus_probe |
| **GP-118** |  | *Moraxella*_Genus_probe_1 |
| **GP-057** |  | *Moraxella*_Genus_probe_2 |
| **GP-058** |  | *Mycobacterium*_Genus_probe |
| **GP-059** |  | *Mycoplasma*_Genus_probe |
| **GP-119** |  | *Neisseria*_Genus_probe_1 |
| **GP-060** |  | *Neisseria*_Genus_probe_2 |
| **GP-061** |  | *Olsenella*_Genus_probe |
| **GP-120** |  | *Oribacterium*_Genus_probe |
| **GP-062** |  | *Paenibacillus*_Genus_probe |
| **GP-063** |  | *Parvimonas*_Genus_probe |
| **GP-064** |  | *Peptoniphilus*_Genus_probe |
| **GP-065** |  | *Peptostreptococcus*_Genus_probe |
| **GP-066** |  | *Porphyromonas*_Genus_probe_1 |
| **GP-067** |  | *Porphyromonas*_Genus_probe_2 |
| **GP-068** |  | *Porphyromonas*_Genus_probe_3 |
| **GP-121** |  | *Prevotella*_Genus_probe_1 |
| **GP-069** |  | *Prevotella*_Genus_probe_2 |
| **GP-070** |  | *Propionibacterium*_Genus_probe |
| **GP-071** |  | *Proteus*_Genus_probe |
| **GP-072** |  | *Pseudomonas*_Genus_probe |
| **GP-073** |  | *Rothia*_Genus_probe |
| **GP-074** |  | *Sanguibacter*_Genus_probe |
| **GP-075** |  | *Scardovia*_Genus_probe |
| **GP-076** |  | *Selenomonas*_&_*Centipeda*_Genus_probe |
| **GP-122** |  | *Selenomonas*_Genus_probe_1 |
| **GP-123** |  | *Selenomonas*_Genus_probe_2 |
| **GP-077** |  | *Slackia*_Genus_probe |
| **GP-078** |  | *Sphingomonas*_Genus_probe |
| **GP-079** |  | SR1_Genus_probe |
| **GP-124** |  | *Staphylococcus*_Genus_probe_1 |
| **GP-125** |  | *Staphylococcus*_Genus_probe_2 |
| **GP-080** |  | *Staphylococcus*_Genus_probe_3 |
| **GP-126** |  | *Streptococcus*_Genus_probe_1 |
| **GP-127** |  | *Streptococcus*_Genus_probe_2 |
| **GP-128** |  | *Streptococcus*_Genus_probe_3 |
| **GP-081** |  | *Streptococcus*_Genus_probe_4 |
| **GP-082** |  | *Tannerella*_Genus_probe |
| **GP-083** |  | TM7_Genus_probe |
| **GP-129** |  | *Treponema*_Genus_probe_1 |
| **GP-084** |  | *Treponema*_Genus_probe_2 |
| **GP-085** |  | *Treponema*_Genus_probe_3 |
| **GP-086** |  | *Treponema*_Genus_probe_4 |
| **GP-087** |  | *Treponema*_Genus_probe_5 |
| **GP-088** |  | *Treponema*_Genus_probe_6 |
| **GP-130** |  | *Veillonella*_Genus_probe_1 |
| **GP-089** |  | *Veillonella*_Genus_probe_2 |
| **GP-131** |  | *Veillonellaceae*_Genus_probe_1 |
| **GP-091** |  | *Veillonellaceae*_Genus_probe_2 |
| **GP-090** |  | *Veillonellaceae*_Genus_probe_3 |
| **GP-092** |  | *Yersinia*_Genus_probe |
