# Supplementary material for: Temporal Stability of the Salivary Microbiota in Oral Health
Source: PLoS One. 2016 Jan 22;11(1):e0147472. doi: 10.1371/journal.pone.0147472 (PMC4723053; doi:10.1371/journal.pone.0147472)
Supplement: S2 File — Complete list of the 477 probes identified (399 recognizing a bacterial taxon and 78 recognizing a bacterial genus). Probes are listed according to their proportional presence (%) across samples in decreasing order. (DOCX) [file pone.0147472.s002.docx]

|  | **Participant 1** |  | **Participant 2** |  | **Participant 3** |  | **Participant 4** |  | **Participant 5** |  |
| --- | --- | --- | --- | --- | --- | --- | --- | --- | --- | --- |
| **Organism Name** | **Presence frequency (%)** | **Proportional presence (%)** | **Presence frequency (%)** | **Proportional presence (%)** | **Presence frequency (%)** | **Proportional presence (%)** | **Presence frequency (%)** | **Proportional presence (%)** | **Presence frequency (%)** | **Proportional presence (%)** |
| ***Streptococcus*_Genus_probe_4** | 100.0 | 26.20389 | 100.0 | 18.58174 | 100.0 | 25.26272 | 100.0 | 23.96497 | 100.0 | 21.93904 |
| ***Haemophilus_parainfluenzae*** | 100.0 | 9.56702 | 100.0 | 9.30773 | 100.0 | 5.17675 | 100.0 | 7.07255 | 100.0 | 4.46353 |
| ***Prevotella_melaninogenica*** | 100.0 | 5.96449 | 100.0 | 8.59056 | 100.0 | 7.01513 | 100.0 | 6.01460 | 100.0 | 4.80850 |
| ***Rothia_mucilaginosa*** | 100.0 | 5.46647 | 100.0 | 8.41174 | 100.0 | 7.12756 | 100.0 | 5.36373 | 100.0 | 5.04618 |
| ***Neisseria*_Genus_probe_2** | 100.0 | 9.76208 | 100.0 | 2.86644 | 100.0 | 1.09216 | 100.0 | 13.43868 | 100.0 | 1.42732 |
| ***Fusobacterium_periodonticum*** | 100.0 | 1.40337 | 100.0 | 4.82267 | 100.0 | 2.94850 | 100.0 | 5.27307 | 100.0 | 9.29682 |
| ***Neisseria_flavescens*** | 100.0 | 1.88541 | 100.0 | 3.80088 | 100.0 | 0.16211 | 100.0 | 1.43651 | 100.0 | 7.76081 |
| ***Porphyromonas*_sp_oral_taxon_279** | 100.0 | 1.44813 | 100.0 | 2.95688 | 100.0 | 0.46989 | 100.0 | 4.34034 | 100.0 | 2.55799 |
| ***Prevotella*_Genus_probe_1** | 100.0 | 1.35681 | 100.0 | 2.21238 | 100.0 | 1.75599 | 100.0 | 2.43263 | 100.0 | 2.12928 |
| ***Neisseria_subflava*** | 75.0 | 0.00282 | 58.3 | 0.00187 | 100.0 | 9.37845 | 100.0 | 0.44837 | 50.0 | 0.00098 |
| ***Granulicatella*_Genus_probe** | 100.0 | 1.38129 | 100.0 | 2.32833 | 100.0 | 1.75844 | 100.0 | 1.25657 | 100.0 | 0.67797 |
| ***Fusobacterium*_Genus_probe_4** | 100.0 | 0.38347 | 100.0 | 0.68556 | 100.0 | 0.78332 | 100.0 | 0.80437 | 100.0 | 3.91815 |
| ***Veillonella*_Genus_probe_2** | 100.0 | 0.68352 | 100.0 | 1.79768 | 100.0 | 1.14179 | 100.0 | 0.85409 | 100.0 | 1.12021 |
| ***Veillonella_rogosae*** | 100.0 | 0.56207 | 100.0 | 1.19643 | 100.0 | 0.99386 | 100.0 | 1.28282 | 100.0 | 0.67915 |
| ***Gemella_haemolysans*** | 100.0 | 0.72255 | 100.0 | 0.51234 | 100.0 | 0.47142 | 100.0 | 0.43016 | 100.0 | 1.83972 |
| ***Granulicatella_elegans*** | 100.0 | 0.16406 | 100.0 | 0.27383 | 100.0 | 0.67838 | 100.0 | 0.22955 | 100.0 | 2.13905 |
| ***Gemella_sanguinis*** | 100.0 | 0.88485 | 100.0 | 1.32967 | 100.0 | 0.31675 | 100.0 | 0.37918 | 100.0 | 0.34312 |
| ***Haemophilus*_Genus_probe_3** | 100.0 | 1.04052 | 100.0 | 0.22862 | 100.0 | 0.75520 | 91.7 | 0.06457 | 100.0 | 0.62826 |
| ***Leptotrichia_*sp_oral_taxon_417** | 100.0 | 0.31060 | 100.0 | 1.83522 | 100.0 | 0.30476 | 100.0 | 0.11423 | 100.0 | 0.12703 |
| ***Haemophilus_parahaemolyticus*** | 100.0 | 0.48755 | 91.7 | 0.02087 | 100.0 | 0.93466 | 41.7 | 0.00292 | 100.0 | 1.01761 |
| ***Oribacterium_sinus*** | 100.0 | 0.35290 | 100.0 | 0.33410 | 100.0 | 0.41469 | 100.0 | 0.32811 | 100.0 | 0.53364 |
| ***Alloprevotella*_sp_oral_taxon_473** | 100.0 | 0.14848 | 100.0 | 0.28919 | 100.0 | 0.10916 | 25.0 | 0.00077 | 100.0 | 1.37439 |
| ***Rothia*_Genus_probe** | 100.0 | 0.59151 | 100.0 | 0.32451 | 100.0 | 0.34302 | 100.0 | 0.26668 | 100.0 | 0.34531 |
| ***Prevotella_pallens*** | 100.0 | 0.19940 | 100.0 | 0.87210 | 100.0 | 0.40609 | 100.0 | 0.25970 | 100.0 | 0.09973 |
| ***Rothia_dentocariosa*** | 100.0 | 0.79836 | 100.0 | 0.13332 | 100.0 | 0.20590 | 100.0 | 0.15363 | 100.0 | 0.50245 |
| ***Leptotrichia*_sp_oral_taxon_215** | 100.0 | 0.25866 | 100.0 | 0.52580 | 100.0 | 0.20252 | 100.0 | 0.17914 | 100.0 | 0.62289 |
| ***Prevotella_salivae*** | 100.0 | 0.18640 | 100.0 | 0.78901 | 100.0 | 0.30790 | 100.0 | 0.19149 | 100.0 | 0.07055 |
| ***Lautropia_mirabilis*** | 100.0 | 0.78809 | 100.0 | 0.05637 | 100.0 | 0.25239 | 100.0 | 0.19823 | 100.0 | 0.13640 |
| ***Campylobacter_concisus*** | 100.0 | 0.36706 | 100.0 | 0.36723 | 100.0 | 0.18503 | 100.0 | 0.28092 | 100.0 | 0.19933 |
| ***Bergeyella*_sp_oral_taxon_322** | 100.0 | 0.62473 | 100.0 | 0.18296 | 100.0 | 0.16219 | 100.0 | 0.16038 | 100.0 | 0.24280 |
| ***Streptococcus*_Genus_probe_1** | 100.0 | 0.23624 | 100.0 | 0.09521 | 100.0 | 0.28984 | 100.0 | 0.13874 | 100.0 | 0.47538 |
| ***Streptococcus_sanguinis*** | 100.0 | 0.42596 | 100.0 | 0.11073 | 100.0 | 0.39319 | 100.0 | 0.21302 | 100.0 | 0.03819 |
| ***Alloprevotella*_sp_oral_taxon_914** | 100.0 | 0.16056 | 100.0 | 0.25100 | 100.0 | 0.12839 | 100.0 | 0.33033 | 100.0 | 0.30460 |
| ***Leptotrichia*_sp_oral_taxon_221** | 100.0 | 0.16957 | 100.0 | 0.59451 | 91.7 | 0.01355 | 100.0 | 0.20547 | 100.0 | 0.18389 |
| ***Prevotella_histicola*** | 100.0 | 0.05013 | 100.0 | 0.88487 | 100.0 | 0.06104 | 100.0 | 0.00744 | 100.0 | 0.07114 |
| ***Rothia_aeria*** | 100.0 | 0.72783 | 100.0 | 0.05508 | 100.0 | 0.11868 | 100.0 | 0.09026 | 100.0 | 0.07411 |
| ***Veillonella_atypica*** | 100.0 | 0.19907 | 100.0 | 0.18587 | 100.0 | 0.27548 | 100.0 | 0.34995 | 100.0 | 0.01442 |
| ***Actinomyces*_Genus_probe_4** | 100.0 | 0.03693 | 100.0 | 0.19356 | 100.0 | 0.37592 | 100.0 | 0.20874 | 100.0 | 0.07672 |
| ***Alloprevotella_tannerae*** | 100.0 | 0.07456 | 100.0 | 0.17461 | 100.0 | 0.01060 | 91.7 | 0.01675 | 100.0 | 0.58518 |
| ***Alloprevotella*_Genus_probe** | 100.0 | 0.06308 | 100.0 | 0.13110 | 100.0 | 0.03798 | 100.0 | 0.03456 | 100.0 | 0.58794 |
| ***Gemella*_Genus_probe** | 100.0 | 0.28040 | 100.0 | 0.19214 | 100.0 | 0.07623 | 100.0 | 0.05840 | 100.0 | 0.23460 |
| ***Lachnoanaerobaculum_umeaense*** | 100.0 | 0.11797 | 100.0 | 0.20992 | 100.0 | 0.11145 | 100.0 | 0.15417 | 100.0 | 0.15893 |
| ***Clostridiales*[F-2][G-1]_sp_oral_taxon_075** | 100.0 | 0.23952 | 100.0 | 0.31331 | 100.0 | 0.01617 | 91.7 | 0.02865 | 100.0 | 0.11371 |
| ***Catonella*_Genus_probe** | 100.0 | 0.07960 | 100.0 | 0.23354 | 100.0 | 0.08961 | 100.0 | 0.09739 | 100.0 | 0.20223 |
| ***Haemophilus*_sp_oral_taxon_035** | 100.0 | 0.32846 | 100.0 | 0.32998 | 16.7 | 0.00031 | 16.7 | 0.00042 | 8.3 | 0.00021 |
| ***Oribacterium*_sp_oral_taxon_108** | 8.3 | 0.00017 | 100.0 | 0.23025 | 100.0 | 0.07353 | 100.0 | 0.08399 | 100.0 | 0.26912 |
| ***Neisseria_elongata*** | 100.0 | 0.14304 | 83.3 | 0.00695 | 100.0 | 0.44627 | 100.0 | 0.03926 | 100.0 | 0.01456 |
| **TM7[G-1]_sp_oral_taxon_352** | 100.0 | 0.06154 | 100.0 | 0.17005 | 100.0 | 0.05556 | 100.0 | 0.13103 | 100.0 | 0.21450 |
| ***Parvimonas*_Genus_probe** | 100.0 | 0.01882 | 100.0 | 0.05731 | 66.7 | 0.00350 | 83.3 | 0.01140 | 100.0 | 0.49848 |
| ***Stomatobaculum*_sp_oral_taxon_097** | 75.0 | 0.00312 | 100.0 | 0.35875 | 100.0 | 0.04755 | 91.7 | 0.00313 | 100.0 | 0.15903 |
| ***Leptotrichia*_Genus_probe_3** | 100.0 | 0.05908 | 100.0 | 0.32883 | 100.0 | 0.06592 | 100.0 | 0.02300 | 100.0 | 0.04058 |
| ***Haemophilus*_Genus_probe_2** | 100.0 | 0.39622 | 75.0 | 0.01157 | 8.3 | 0.00018 | 100.0 | 0.08279 | 91.7 | 0.02447 |
| ***Campylobacter*_Genus_probe_2** | 100.0 | 0.12714 | 100.0 | 0.12995 | 100.0 | 0.07084 | 100.0 | 0.10275 | 100.0 | 0.06921 |
| ***Neisseria_pharyngis*** | 16.7 | 0.00032 | 33.3 | 0.00103 | 100.0 | 0.46598 | 91.7 | 0.01295 | 33.3 | 0.00091 |
| ***Leptotrichia*_Genus_probe_4** | 100.0 | 0.08039 | 100.0 | 0.20612 | 100.0 | 0.02685 | 100.0 | 0.04743 | 100.0 | 0.06016 |
| ***Aggregatibacter*_Genus_probe_2** | 100.0 | 0.24700 | 100.0 | 0.04667 | 33.3 | 0.00075 | 50.0 | 0.00232 | 100.0 | 0.12089 |
| ***Solobacterium_moorei*** | 100.0 | 0.06844 | 100.0 | 0.13928 | 100.0 | 0.05658 | 100.0 | 0.05932 | 100.0 | 0.07883 |
| ***Aggregatibacter*_Genus_probe_1** | 100.0 | 0.04220 | 100.0 | 0.02909 | 100.0 | 0.21361 | 100.0 | 0.08993 | 100.0 | 0.01310 |
| ***Veillonella_dispar*** | 100.0 | 0.11964 | 100.0 | 0.06492 | 100.0 | 0.10002 | 100.0 | 0.07657 | 91.7 | 0.00728 |
| ***Prevotella_intermedia*** | 100.0 | 0.23603 | 100.0 | 0.12936 | 8.3 | 0.00010 | 0.0 | 0.00000 | 8.3 | 0.00014 |
| **SR1[G-1]_sp_oral_taxon_875** | 16.7 | 0.00035 | 91.7 | 0.08333 | 66.7 | 0.00379 | 100.0 | 0.10139 | 100.0 | 0.16221 |
| ***Veillonella_atypica*** | 100.0 | 0.09097 | 100.0 | 0.09876 | 91.7 | 0.04316 | 100.0 | 0.11718 | 33.3 | 0.00089 |
| ***Veillonella*_sp_oral_taxon_780** | 100.0 | 0.14144 | 91.7 | 0.07313 | 100.0 | 0.01846 | 33.3 | 0.00072 | 100.0 | 0.11333 |
| ***Prevotella_pallens*** | 100.0 | 0.06695 | 100.0 | 0.23795 | 100.0 | 0.01010 | 91.7 | 0.00562 | 100.0 | 0.00996 |
| ***Prevotella_scopos*** | 0.0 | 0.00000 | 100.0 | 0.27679 | 8.3 | 0.00021 | 8.3 | 0.00027 | 100.0 | 0.05149 |
| ***Neisseria_pharyngis*** | 8.3 | 0.00026 | 8.3 | 0.00016 | 100.0 | 0.08597 | 100.0 | 0.11043 | 100.0 | 0.12508 |
| ***Clostridiales*[F-2][G-2]_sp_oral_taxon_085** | 100.0 | 0.04318 | 100.0 | 0.10649 | 100.0 | 0.01285 | 100.0 | 0.03596 | 100.0 | 0.10699 |
| ***Peptostreptococcus_stomatis*** | 100.0 | 0.02707 | 100.0 | 0.04892 | 100.0 | 0.06656 | 100.0 | 0.04013 | 100.0 | 0.12231 |
| ***Aggregatibacter_paraphrophilus*** | 100.0 | 0.10668 | 0.0 | 0.00000 | 100.0 | 0.11970 | 100.0 | 0.03210 | 100.0 | 0.02692 |
| ***Selenomonas*_sp_oral_taxon_149** | 91.7 | 0.02600 | 100.0 | 0.18564 | 91.7 | 0.01043 | 100.0 | 0.03878 | 83.3 | 0.01288 |
| ***Actinomyces_odontolyticus*** | 75.0 | 0.00824 | 100.0 | 0.14360 | 100.0 | 0.09530 | 100.0 | 0.00787 | 83.3 | 0.01151 |
| ***Prevotella_oris*** | 100.0 | 0.10884 | 100.0 | 0.05145 | 100.0 | 0.01991 | 91.7 | 0.01444 | 100.0 | 0.06094 |
| **TM7_Genus_probe** | 100.0 | 0.10284 | 100.0 | 0.02925 | 100.0 | 0.02413 | 100.0 | 0.06915 | 100.0 | 0.02986 |
| ***Leptotrichia_hongkongensis*** | 100.0 | 0.11825 | 100.0 | 0.06376 | 100.0 | 0.01246 | 100.0 | 0.05573 | 25.0 | 0.00121 |
| ***Lachnoanaerobaculum*_sp_oral_taxon_083** | 16.7 | 0.00024 | 100.0 | 0.02005 | 25.0 | 0.00039 | 25.0 | 0.00055 | 100.0 | 0.22161 |
| ***Oribacterium*_sp_oral_taxon_108** | 100.0 | 0.05312 | 100.0 | 0.05007 | 100.0 | 0.05116 | 100.0 | 0.06240 | 91.7 | 0.01781 |
| ***Simonsiella_muelleri*** | 0.0 | 0.00000 | 100.0 | 0.05071 | 100.0 | 0.06689 | 91.7 | 0.07336 | 100.0 | 0.04119 |
| ***Capnocytophaga_sputigena*** | 100.0 | 0.09811 | 100.0 | 0.01670 | 91.7 | 0.00507 | 75.0 | 0.00292 | 100.0 | 0.10432 |
| ***Actinomyces*_sp_oral_taxon_172** | 91.7 | 0.04513 | 91.7 | 0.00811 | 100.0 | 0.05558 | 100.0 | 0.06455 | 100.0 | 0.04628 |
| ***Prevotella*_Genus_probe_2** | 100.0 | 0.07915 | 100.0 | 0.05907 | 100.0 | 0.02113 | 100.0 | 0.01974 | 91.7 | 0.03937 |
| ***Aggregatibacter*_sp_oral_taxon_458** | 16.7 | 0.00032 | 100.0 | 0.11817 | 100.0 | 0.09043 | 25.0 | 0.00082 | 66.7 | 0.00332 |
| ***Corynebacterium_durum*** | 100.0 | 0.01896 | 100.0 | 0.02295 | 100.0 | 0.11880 | 100.0 | 0.02954 | 91.7 | 0.02222 |
| ***Lachnospiraceae*[G-5]_sp_oral_taxon_455** | 83.3 | 0.01588 | 100.0 | 0.15127 | 100.0 | 0.01780 | 75.0 | 0.00910 | 91.7 | 0.01638 |
| ***Porphyromonas*_Genus_probe_2** | 100.0 | 0.09686 | 100.0 | 0.07726 | 100.0 | 0.02111 | 83.3 | 0.00601 | 83.3 | 0.00590 |
| ***Prevotella_veroralis*** | 41.7 | 0.00162 | 75.0 | 0.00694 | 100.0 | 0.11293 | 100.0 | 0.07831 | 91.7 | 0.00688 |
| ***Selenomonas*_sp_oral_taxon_136** | 100.0 | 0.02021 | 100.0 | 0.15708 | 58.3 | 0.00388 | 91.7 | 0.00537 | 75.0 | 0.00375 |
| ***Prevotella*_sp_oral_taxon_309** | 83.3 | 0.01792 | 100.0 | 0.14525 | 75.0 | 0.00571 | 91.7 | 0.01584 | 8.3 | 0.00021 |
| ***Actinomyces_odontolyticus*** | 100.0 | 0.07838 | 100.0 | 0.03513 | 100.0 | 0.02065 | 83.3 | 0.00315 | 100.0 | 0.04422 |
| ***Streptococcus*_sp_oral_taxon_064** | 100.0 | 0.04051 | 100.0 | 0.02535 | 100.0 | 0.04152 | 100.0 | 0.03729 | 100.0 | 0.03557 |
| ***Eubacterium*[11][G-1]_sulci** | 91.7 | 0.00714 | 100.0 | 0.09834 | 100.0 | 0.03946 | 100.0 | 0.01190 | 100.0 | 0.02339 |
| ***Abiotrophia_defectiva*** | 100.0 | 0.08632 | 100.0 | 0.02410 | 100.0 | 0.03093 | 100.0 | 0.01920 | 91.7 | 0.01117 |
| ***Fusobacterium_*Genus_probe_2** | 100.0 | 0.07405 | 100.0 | 0.05650 | 91.7 | 0.01019 | 100.0 | 0.00960 | 100.0 | 0.01716 |
| ***Megasphaera_micronuciformis*** | 100.0 | 0.03642 | 100.0 | 0.06493 | 91.7 | 0.01854 | 100.0 | 0.02453 | 100.0 | 0.02231 |
| ***Eikenella_corrodens*** | 100.0 | 0.05025 | 91.7 | 0.00689 | 100.0 | 0.00672 | 83.3 | 0.00489 | 100.0 | 0.08278 |
| ***Kingella_oralis*** | 100.0 | 0.08645 | 83.3 | 0.01107 | 100.0 | 0.02812 | 100.0 | 0.01108 | 100.0 | 0.01334 |
| ***Kingella*_Genus_probe_1** | 100.0 | 0.04439 | 100.0 | 0.01756 | 58.3 | 0.00152 | 91.7 | 0.01581 | 100.0 | 0.06803 |
| ***Prevotella_oulorum*** | 100.0 | 0.08581 | 83.3 | 0.01261 | 100.0 | 0.02763 | 100.0 | 0.01825 | 33.3 | 0.00088 |
| ***Alloprevotella*_sp_oral_taxon_914** | 0.0 | 0.00000 | 16.7 | 0.00051 | 91.7 | 0.03450 | 100.0 | 0.10298 | 8.3 | 0.00015 |
| ***Corynebacterium_matruchotii*** | 100.0 | 0.04169 | 100.0 | 0.01811 | 100.0 | 0.05635 | 91.7 | 0.01606 | 66.7 | 0.00185 |
| **SR1[G-1]_sp_oral_taxon_345** | 91.7 | 0.02022 | 91.7 | 0.03909 | 41.7 | 0.00070 | 41.7 | 0.00187 | 100.0 | 0.07084 |
| ***Oribacterium*_sp_oral_taxon_108** | 33.3 | 0.00105 | 100.0 | 0.05076 | 100.0 | 0.04458 | 100.0 | 0.01642 | 100.0 | 0.01947 |
| ***Lachnoanaerobaculum_orale*** | 91.7 | 0.02667 | 100.0 | 0.07481 | 91.7 | 0.01963 | 100.0 | 0.00705 | 58.3 | 0.00216 |
| ***Capnocytophaga_leadbetteri*** | 100.0 | 0.03116 | 100.0 | 0.06045 | 100.0 | 0.02138 | 91.7 | 0.00918 | 83.3 | 0.00775 |
| ***Streptococcus_parasanguinis*_II** | 100.0 | 0.02990 | 100.0 | 0.02273 | 100.0 | 0.02433 | 100.0 | 0.02953 | 100.0 | 0.02047 |
| ***Veillonella_denticariosi*** | 0.0 | 0.00000 | 100.0 | 0.12311 | 41.7 | 0.00156 | 8.3 | 0.00032 | 8.3 | 0.00042 |
| ***Streptococcus_*sp_oral_taxon_066** | 100.0 | 0.03001 | 100.0 | 0.01939 | 100.0 | 0.02191 | 100.0 | 0.02704 | 100.0 | 0.02294 |
| **TM7[G-3]_sp_oral_taxon_351** | 100.0 | 0.06131 | 100.0 | 0.02012 | 100.0 | 0.00632 | 91.7 | 0.01042 | 100.0 | 0.01801 |
| ***Leptotrichia_goodfellowii*** | 100.0 | 0.09660 | 75.0 | 0.00749 | 100.0 | 0.00395 | 50.0 | 0.00197 | 66.7 | 0.00369 |
| ***Neisseria*_Genus_probe_1** | 0.0 | 0.00000 | 8.3 | 0.00015 | 25.0 | 0.00047 | 100.0 | 0.09276 | 83.3 | 0.01976 |
| ***Alloprevotella*_sp_oral_taxon_308** | 100.0 | 0.01413 | 100.0 | 0.05115 | 100.0 | 0.02718 | 100.0 | 0.02038 | 0.0 | 0.00000 |
| ***Veillonella*_Genus_probe_1** | 100.0 | 0.02175 | 100.0 | 0.01966 | 100.0 | 0.04328 | 100.0 | 0.02495 | 25.0 | 0.00111 |
| ***Veillonella_parvula*** | 100.0 | 0.04016 | 100.0 | 0.01472 | 100.0 | 0.02801 | 100.0 | 0.01295 | 100.0 | 0.01467 |
| ***Fusobacterium*_Genus_probe_3** | 100.0 | 0.03478 | 100.0 | 0.02211 | 100.0 | 0.01627 | 83.3 | 0.00624 | 100.0 | 0.02815 |
| ***Actinomyces_*sp_oral_taxon_181** | 50.0 | 0.00160 | 100.0 | 0.01658 | 100.0 | 0.02911 | 100.0 | 0.05672 | 66.7 | 0.00211 |
| ***Prevotella_shahii*** | 58.3 | 0.01119 | 100.0 | 0.03893 | 8.3 | 0.00012 | 91.7 | 0.00659 | 100.0 | 0.04487 |
| ***Aggregatibacter*_sp_oral_taxon_513** | 100.0 | 0.02518 | 66.7 | 0.00490 | 100.0 | 0.03691 | 91.7 | 0.01206 | 100.0 | 0.02059 |
| ***Streptococcus_intermedius*** | 100.0 | 0.01757 | 100.0 | 0.02411 | 100.0 | 0.01867 | 100.0 | 0.02999 | 91.7 | 0.00917 |
| ***Cardiobacterium_hominis*** | 100.0 | 0.06284 | 66.7 | 0.00414 | 100.0 | 0.01889 | 100.0 | 0.00935 | 58.3 | 0.00355 |
| ***Porphyromonas*_sp_oral_taxon_395** | 100.0 | 0.07791 | 91.7 | 0.02058 | 0.0 | 0.00000 | 0.0 | 0.00000 | 8.3 | 0.00014 |
| ***Peptostreptococcaceae*[11][G-7]_sp_oral_taxon_106** | 100.0 | 0.05806 | 8.3 | 0.00024 | 100.0 | 0.03670 | 25.0 | 0.00069 | 0.0 | 0.00000 |
| ***Streptococcus*_sp_oral_taxon_486** | 100.0 | 0.02033 | 100.0 | 0.01380 | 100.0 | 0.01542 | 100.0 | 0.02693 | 100.0 | 0.01312 |
| ***Streptococcus*_sp_oral_taxon_431** | 100.0 | 0.01935 | 100.0 | 0.01735 | 100.0 | 0.02027 | 100.0 | 0.01490 | 100.0 | 0.01702 |
| ***Gemella_morbillorum*** | 100.0 | 0.02553 | 91.7 | 0.01419 | 100.0 | 0.03062 | 100.0 | 0.00633 | 100.0 | 0.01148 |
| ***Leptotrichia_wadei*** | 100.0 | 0.04326 | 91.7 | 0.00939 | 100.0 | 0.02853 | 16.7 | 0.00028 | 58.3 | 0.00627 |
| ***Actinomyces_graevenitzii*** | 75.0 | 0.01288 | 100.0 | 0.02536 | 100.0 | 0.03518 | 0.0 | 0.00000 | 91.7 | 0.01224 |
| ***Leptotrichia*_sp_oral_taxon_392** | 100.0 | 0.03270 | 100.0 | 0.02417 | 100.0 | 0.01274 | 100.0 | 0.01026 | 75.0 | 0.00411 |
| ***Porphyromonas*_sp_oral_taxon_279** | 58.3 | 0.00148 | 100.0 | 0.01729 | 58.3 | 0.00201 | 100.0 | 0.01992 | 100.0 | 0.04300 |
| ***Dialister_invisus*** | 100.0 | 0.03976 | 100.0 | 0.01362 | 83.3 | 0.00653 | 91.7 | 0.01224 | 91.7 | 0.00949 |
| ***Leptotrichia*_sp_oral_taxon_223** | 100.0 | 0.05011 | 100.0 | 0.02508 | 25.0 | 0.00058 | 41.7 | 0.00184 | 91.7 | 0.00365 |
| ***Prevotella_nigrescens*** | 91.7 | 0.01438 | 100.0 | 0.02845 | 83.3 | 0.01323 | 100.0 | 0.01050 | 83.3 | 0.01401 |
| ***Campylobacter_gracilis*** | 100.0 | 0.03937 | 100.0 | 0.01920 | 83.3 | 0.01084 | 91.7 | 0.00523 | 66.7 | 0.00394 |
| ***Actinomyces_*Genus_probe_3** | 100.0 | 0.00858 | 91.7 | 0.00617 | 100.0 | 0.03497 | 100.0 | 0.02115 | 66.7 | 0.00441 |
| ***Porphyromonas_endodontalis*** | 100.0 | 0.04671 | 0.0 | 0.00000 | 41.7 | 0.00145 | 91.7 | 0.01046 | 91.7 | 0.01498 |
| ***Leptotrichia*_sp_oral_taxon_212** | 100.0 | 0.03663 | 100.0 | 0.01583 | 100.0 | 0.01194 | 91.7 | 0.00474 | 83.3 | 0.00339 |
| ***Mogibacterium*_Genus_probe** | 91.7 | 0.00975 | 100.0 | 0.02396 | 100.0 | 0.01851 | 83.3 | 0.00634 | 91.7 | 0.01063 |
| ***Selenomonas*_&_*Centipeda*_Genus_probe** | 100.0 | 0.05361 | 66.7 | 0.00545 | 75.0 | 0.00532 | 33.3 | 0.00081 | 41.7 | 0.00193 |
| ***Capnocytophaga_gingivalis*** | 100.0 | 0.01417 | 100.0 | 0.01544 | 100.0 | 0.01474 | 91.7 | 0.00909 | 100.0 | 0.01354 |
| ***Treponema*_Genus_probe_2** | 33.3 | 0.00285 | 100.0 | 0.01978 | 33.3 | 0.00086 | 33.3 | 0.00086 | 100.0 | 0.04261 |
| ***Parvimonas_micra*** | 100.0 | 0.01815 | 100.0 | 0.02975 | 83.3 | 0.00450 | 91.7 | 0.00541 | 66.7 | 0.00682 |
| ***Prevotella_denticola*** | 100.0 | 0.02831 | 100.0 | 0.00900 | 91.7 | 0.01181 | 91.7 | 0.01096 | 83.3 | 0.00348 |
| ***Lachnospiraceae*[G-2]_sp_oral_taxon_096** | 41.7 | 0.00233 | 100.0 | 0.04673 | 8.3 | 0.00015 | 33.3 | 0.00144 | 91.7 | 0.01289 |
| ***Stomatobaculum_longum*** | 100.0 | 0.03237 | 41.7 | 0.00109 | 50.0 | 0.00387 | 75.0 | 0.00442 | 100.0 | 0.02028 |
| ***Campylobacter*_sp_oral_taxon_044** | 0.0 | 0.00000 | 100.0 | 0.02316 | 75.0 | 0.00459 | 75.0 | 0.01161 | 100.0 | 0.02134 |
| ***Prevotella_maculosa*** | 100.0 | 0.03850 | 100.0 | 0.00905 | 83.3 | 0.00933 | 58.3 | 0.00205 | 58.3 | 0.00151 |
| ***Streptococcus*_Genus_probe_3** | 100.0 | 0.00903 | 75.0 | 0.00641 | 91.7 | 0.01301 | 100.0 | 0.02177 | 91.7 | 0.00930 |
| ***Kingella*_sp_oral_taxon_459** | 100.0 | 0.02151 | 83.3 | 0.00486 | 0.0 | 0.00000 | 100.0 | 0.02966 | 66.7 | 0.00245 |
| ***Porphyromonas*_sp_oral_taxon_275** | 100.0 | 0.03579 | 100.0 | 0.01084 | 100.0 | 0.00711 | 75.0 | 0.00426 | 0.0 | 0.00000 |
| **TM7[G-1]_sp_oral_taxon_346** | 100.0 | 0.05371 | 25.0 | 0.00063 | 41.7 | 0.00198 | 16.7 | 0.00030 | 25.0 | 0.00067 |
| ***Campylobacter_concisus*** | 100.0 | 0.01961 | 91.7 | 0.00967 | 91.7 | 0.02125 | 58.3 | 0.00199 | 66.7 | 0.00229 |
| ***Atopobium*_Genus_probe** | 100.0 | 0.01607 | 100.0 | 0.01829 | 91.7 | 0.01104 | 66.7 | 0.00297 | 100.0 | 0.00504 |
| ***Capnocytophaga_granulosa*** | 100.0 | 0.02160 | 100.0 | 0.00745 | 83.3 | 0.00514 | 25.0 | 0.00041 | 100.0 | 0.01691 |
| ***Cardiobacterium_valvarum*** | 100.0 | 0.04391 | 58.3 | 0.00246 | 66.7 | 0.00325 | 0.0 | 0.00000 | 25.0 | 0.00056 |
| ***Porphyromonas*_sp_oral_taxon_278** | 33.3 | 0.00062 | 91.7 | 0.01107 | 58.3 | 0.00142 | 91.7 | 0.00998 | 100.0 | 0.02587 |
| ***Dialister_pneumosintes*** | 100.0 | 0.02336 | 100.0 | 0.01537 | 0.0 | 0.00000 | 0.0 | 0.00000 | 75.0 | 0.00989 |
| ***Prevotella_baroniae*** | 100.0 | 0.01459 | 100.0 | 0.02174 | 50.0 | 0.00144 | 75.0 | 0.00879 | 0.0 | 0.00000 |
| ***Leptotrichia*_sp_oral_taxon_219** | 100.0 | 0.02523 | 100.0 | 0.01651 | 25.0 | 0.00041 | 83.3 | 0.00378 | 25.0 | 0.00039 |
| ***Treponema*_Genus_probe_6** | 100.0 | 0.02069 | 91.7 | 0.00754 | 33.3 | 0.00055 | 25.0 | 0.00075 | 100.0 | 0.01616 |
| ***Gemella_morbillorum*** | 91.7 | 0.00763 | 91.7 | 0.01237 | 75.0 | 0.00358 | 50.0 | 0.00109 | 100.0 | 0.01975 |
| ***Actinomyces*_sp_oral_taxon_877** | 0.0 | 0.00000 | 100.0 | 0.04395 | 8.3 | 0.00029 | 0.0 | 0.00000 | 0.0 | 0.00000 |
| ***Bacteroidales*[G-2]_sp_oral_taxon_274** | 100.0 | 0.01243 | 91.7 | 0.01588 | 66.7 | 0.00460 | 83.3 | 0.00371 | 83.3 | 0.00409 |
| **SR1_Genus_probe** | 41.7 | 0.00191 | 91.7 | 0.01248 | 50.0 | 0.00129 | 50.0 | 0.00135 | 100.0 | 0.02327 |
| ***Capnocytophaga*_Genus_probe_2** | 100.0 | 0.01802 | 83.3 | 0.00911 | 91.7 | 0.00538 | 75.0 | 0.00261 | 75.0 | 0.00492 |
| ***Alloprevotella_rava*** | 66.7 | 0.00203 | 100.0 | 0.02355 | 16.7 | 0.00045 | 25.0 | 0.00106 | 91.7 | 0.01241 |
| **TM7[G-1]_sp_oral_taxon_347** | 100.0 | 0.03654 | 33.3 | 0.00096 | 25.0 | 0.00059 | 33.3 | 0.00064 | 16.7 | 0.00033 |
| ***Peptococcus*_sp_oral_taxon_167** | 50.0 | 0.00191 | 91.7 | 0.00865 | 100.0 | 0.00870 | 16.7 | 0.00042 | 100.0 | 0.01854 |
| ***Tannerella*_sp_oral_taxon_808** | 66.7 | 0.00262 | 100.0 | 0.01869 | 41.7 | 0.00159 | 75.0 | 0.00241 | 100.0 | 0.01167 |
| ***Eubacterium*[11][G-3]_brachy** | 100.0 | 0.01249 | 100.0 | 0.01828 | 58.3 | 0.00252 | 58.3 | 0.00229 | 25.0 | 0.00134 |
| ***Tannerella*_sp_oral_taxon_286** | 100.0 | 0.02443 | 100.0 | 0.00596 | 83.3 | 0.00432 | 41.7 | 0.00103 | 33.3 | 0.00098 |
| ***Prevotella*_sp_oral_taxon_396** | 0.0 | 0.00000 | 100.0 | 0.01647 | 91.7 | 0.00932 | 58.3 | 0.00182 | 91.7 | 0.00910 |
| ***Fusobacterium_nucleatum*_subsp_nucleatum** | 100.0 | 0.01465 | 91.7 | 0.01279 | 66.7 | 0.00114 | 50.0 | 0.00171 | 66.7 | 0.00574 |
| ***Capnocytophaga*_Genus_probe_3** | 91.7 | 0.01060 | 91.7 | 0.00736 | 91.7 | 0.00713 | 66.7 | 0.00376 | 91.7 | 0.00646 |
| ***Capnocytophaga*_sp_oral_taxon_338** | 91.7 | 0.01540 | 41.7 | 0.00123 | 100.0 | 0.01618 | 58.3 | 0.00116 | 33.3 | 0.00095 |
| ***Leptotrichia*_sp_oral_taxon_879** | 25.0 | 0.00036 | 91.7 | 0.00589 | 100.0 | 0.01761 | 91.7 | 0.00974 | 25.0 | 0.00053 |
| ***Treponema_denticola*** | 100.0 | 0.01339 | 100.0 | 0.01264 | 16.7 | 0.00041 | 0.0 | 0.00000 | 41.7 | 0.00681 |
| ***Streptococcus_constellatus*** | 100.0 | 0.02716 | 75.0 | 0.00470 | 16.7 | 0.00037 | 8.3 | 0.00032 | 8.3 | 0.00015 |
| **SR1[G-1]_sp_oral_taxon_874** | 50.0 | 0.00213 | 100.0 | 0.00665 | 66.7 | 0.00341 | 50.0 | 0.00246 | 100.0 | 0.01747 |
| ***Prevotella*_sp_oral_taxon_472** | 91.7 | 0.01368 | 91.7 | 0.00767 | 50.0 | 0.00440 | 50.0 | 0.00142 | 66.7 | 0.00334 |
| ***Selenomonas*_sp_oral_taxon_478** | 58.3 | 0.00231 | 91.7 | 0.02252 | 0.0 | 0.00000 | 50.0 | 0.00190 | 75.0 | 0.00298 |
| ***Capnocytophaga_haemolytica*** | 100.0 | 0.02932 | 8.3 | 0.00014 | 8.3 | 0.00016 | 0.0 | 0.00000 | 0.0 | 0.00000 |
| ***Selenomonas_noxia*** | 8.3 | 0.00033 | 8.3 | 0.00022 | 91.7 | 0.02855 | 8.3 | 0.00022 | 0.0 | 0.00000 |
| ***Filifactor_alocis*** | 100.0 | 0.01180 | 91.7 | 0.01355 | 0.0 | 0.00000 | 0.0 | 0.00000 | 50.0 | 0.00374 |
| ***Actinobaculum*_sp_oral_taxon_183** | 91.7 | 0.01717 | 91.7 | 0.00813 | 25.0 | 0.00073 | 50.0 | 0.00171 | 16.7 | 0.00025 |
| ***Leptotrichia_shahii*** | 100.0 | 0.02191 | 50.0 | 0.00418 | 0.0 | 0.00000 | 0.0 | 0.00000 | 33.3 | 0.00107 |
| ***Neisseria_meningitidis*** | 91.7 | 0.00814 | 58.3 | 0.00281 | 58.3 | 0.00162 | 91.7 | 0.01197 | 50.0 | 0.00171 |
| ***Fusobacterium_necrophorum*** | 0.0 | 0.00000 | 0.0 | 0.00000 | 0.0 | 0.00000 | 0.0 | 0.00000 | 66.7 | 0.02622 |
| ***Lachnoanaerobaculum_saburreum*** | 100.0 | 0.01674 | 66.7 | 0.00337 | 58.3 | 0.00424 | 16.7 | 0.00037 | 25.0 | 0.00064 |
| ***Selenomonas_dianae*** | 91.7 | 0.02386 | 16.7 | 0.00068 | 8.3 | 0.00010 | 0.0 | 0.00000 | 25.0 | 0.00064 |
| ***Capnocytophaga_ochracea*** | 100.0 | 0.01836 | 0.0 | 0.00000 | 16.7 | 0.00041 | 25.0 | 0.00054 | 66.7 | 0.00530 |
| ***Eubacterium*[11][G-7]_yurii** | 91.7 | 0.02392 | 0.0 | 0.00000 | 0.0 | 0.00000 | 0.0 | 0.00000 | 0.0 | 0.00000 |
| ***Centipeda_periodontii*** | 91.7 | 0.01640 | 58.3 | 0.00495 | 41.7 | 0.00158 | 16.7 | 0.00037 | 33.3 | 0.00059 |
| ***Mycoplasma_orale*** | 0.0 | 0.00000 | 75.0 | 0.00600 | 0.0 | 0.00000 | 0.0 | 0.00000 | 91.7 | 0.01726 |
| ***Mitsuokella*_sp_oral_taxon_131** | 75.0 | 0.02259 | 0.0 | 0.00000 | 0.0 | 0.00000 | 0.0 | 0.00000 | 0.0 | 0.00000 |
| ***Selenomonas*_sp_oral_taxon_134** | 91.7 | 0.01824 | 33.3 | 0.00207 | 0.0 | 0.00000 | 25.0 | 0.00115 | 41.7 | 0.00092 |
| ***Cardiobacterium*_Genus_probe** | 91.7 | 0.00942 | 91.7 | 0.00578 | 58.3 | 0.00253 | 66.7 | 0.00219 | 41.7 | 0.00200 |
| ***Actinomyces_naeslundii*** | 100.0 | 0.01336 | 25.0 | 0.00058 | 83.3 | 0.00687 | 33.3 | 0.00085 | 8.3 | 0.00011 |
| ***Actinomyces_odontolyticus*** | 83.3 | 0.01521 | 0.0 | 0.00000 | 0.0 | 0.00000 | 100.0 | 0.00572 | 0.0 | 0.00000 |
| ***Porphyromonas_catoniae*** | 75.0 | 0.00511 | 83.3 | 0.01223 | 0.0 | 0.00000 | 75.0 | 0.00274 | 41.7 | 0.00083 |
| ***Corynebacterium*_Genus_probe** | 75.0 | 0.00321 | 41.7 | 0.00147 | 100.0 | 0.01048 | 75.0 | 0.00322 | 58.3 | 0.00217 |
| ***Selenomonas_sputigena*** | 50.0 | 0.01408 | 0.0 | 0.00000 | 41.7 | 0.00329 | 75.0 | 0.00249 | 25.0 | 0.00058 |
| **TM7[G-1]_sp_oral_taxon_353** | 58.3 | 0.00404 | 83.3 | 0.00870 | 58.3 | 0.00300 | 75.0 | 0.00316 | 41.7 | 0.00103 |
| ***Tannerella_forsythia*** | 91.7 | 0.00889 | 100.0 | 0.00882 | 25.0 | 0.00061 | 16.7 | 0.00056 | 33.3 | 0.00085 |
| ***Peptostreptococcaceae*[11][G-2]_sp_oral_taxon_091** | 0.0 | 0.00000 | 75.0 | 0.00401 | 83.3 | 0.00459 | 0.0 | 0.00000 | 100.0 | 0.01094 |
| ***Leptotrichia*_sp_oral_taxon_218** | 100.0 | 0.01901 | 0.0 | 0.00000 | 16.7 | 0.00028 | 8.3 | 0.00013 | 0.0 | 0.00000 |
| ***Prevotella*_sp_oral_taxon_315** | 50.0 | 0.00436 | 83.3 | 0.00991 | 25.0 | 0.00061 | 25.0 | 0.00062 | 75.0 | 0.00387 |
| ***Oribacterium*_Genus_probe** | 91.7 | 0.01032 | 50.0 | 0.00303 | 41.7 | 0.00181 | 16.7 | 0.00042 | 58.3 | 0.00380 |
| ***Atopobium_parvulum*** | 50.0 | 0.00593 | 33.3 | 0.00152 | 66.7 | 0.00503 | 50.0 | 0.00169 | 75.0 | 0.00513 |
| ***Actinomyces_johnsonii*** | 100.0 | 0.01096 | 25.0 | 0.00054 | 100.0 | 0.00633 | 8.3 | 0.00032 | 25.0 | 0.00036 |
| ***Mycoplasma_salivarium*** | 83.3 | 0.00702 | 100.0 | 0.00828 | 16.7 | 0.00032 | 16.7 | 0.00043 | 41.7 | 0.00245 |
| ***Treponema_putidum*** | 25.0 | 0.00079 | 91.7 | 0.01644 | 0.0 | 0.00000 | 0.0 | 0.00000 | 8.3 | 0.00046 |
| ***Bergeyella*_sp_oral_taxon_900** | 91.7 | 0.01659 | 8.3 | 0.00031 | 16.7 | 0.00025 | 25.0 | 0.00041 | 0.0 | 0.00000 |
| ***Prevotella_fusca*** | 100.0 | 0.01747 | 0.0 | 0.00000 | 0.0 | 0.00000 | 0.0 | 0.00000 | 0.0 | 0.00000 |
| ***Veillonella_parvula*** | 66.7 | 0.00243 | 100.0 | 0.01466 | 16.7 | 0.00036 | 0.0 | 0.00000 | 0.0 | 0.00000 |
| ***Prevotella_saccharolytica*** | 100.0 | 0.01368 | 0.0 | 0.00000 | 75.0 | 0.00245 | 50.0 | 0.00102 | 16.7 | 0.00028 |
| ***Peptostreptococcus*_Genus_probe** | 41.7 | 0.00146 | 58.3 | 0.00295 | 75.0 | 0.00370 | 75.0 | 0.00331 | 91.7 | 0.00562 |
| ***Eubacterium*_Genus_probe_1** | 50.0 | 0.00219 | 100.0 | 0.00923 | 75.0 | 0.00278 | 41.7 | 0.00097 | 58.3 | 0.00171 |
| ***Selenomonas_noxia*** | 75.0 | 0.01134 | 50.0 | 0.00214 | 0.0 | 0.00000 | 16.7 | 0.00026 | 58.3 | 0.00304 |
| ***Lactococcus_lactis*** | 8.3 | 0.00703 | 0.0 | 0.00000 | 16.7 | 0.00024 | 25.0 | 0.00307 | 25.0 | 0.00543 |
| ***Neisseria*_sp_oral_taxon_499** | 100.0 | 0.01518 | 0.0 | 0.00000 | 0.0 | 0.00000 | 8.3 | 0.00013 | 8.3 | 0.00011 |
| ***Atopobium_parvulum*** | 41.7 | 0.00085 | 91.7 | 0.01310 | 8.3 | 0.00046 | 33.3 | 0.00055 | 16.7 | 0.00032 |
| ***Veillonella_denticariosi*** | 100.0 | 0.00913 | 50.0 | 0.00174 | 75.0 | 0.00224 | 50.0 | 0.00134 | 25.0 | 0.00046 |
| ***Porphyromonas*_sp_oral_taxon_279** | 75.0 | 0.00220 | 58.3 | 0.00484 | 25.0 | 0.00047 | 91.7 | 0.00416 | 75.0 | 0.00293 |
| ***Shuttleworthia_satelles*** | 91.7 | 0.01252 | 41.7 | 0.00159 | 8.3 | 0.00010 | 0.0 | 0.00000 | 16.7 | 0.00035 |
| ***Treponema*_sp_oral_taxon_257** | 100.0 | 0.01017 | 58.3 | 0.00378 | 0.0 | 0.00000 | 0.0 | 0.00000 | 0.0 | 0.00000 |
| ***Fusobacterium_nucleatum*_subsp_polymorphum** | 66.7 | 0.00261 | 83.3 | 0.00560 | 16.7 | 0.00037 | 41.7 | 0.00144 | 66.7 | 0.00367 |
| ***Porphyromonas*_sp_oral_taxon_284** | 58.3 | 0.00263 | 66.7 | 0.00342 | 41.7 | 0.00088 | 83.3 | 0.00346 | 66.7 | 0.00308 |
| ***Selenomonas*_sp_oral_taxon_137** | 33.3 | 0.00386 | 8.3 | 0.00017 | 75.0 | 0.00570 | 75.0 | 0.00216 | 0.0 | 0.00000 |
| ***Prevotella_pleuritidis*** | 100.0 | 0.00509 | 0.0 | 0.00000 | 58.3 | 0.00277 | 0.0 | 0.00000 | 58.3 | 0.00389 |
| ***Lachnoanaerobaculum*_Genus_probe** | 66.7 | 0.00267 | 100.0 | 0.00666 | 50.0 | 0.00166 | 16.7 | 0.00028 | 8.3 | 0.00028 |
| ***Fusobacterium_nucleatum*_subsp_vincentii** | 66.7 | 0.00264 | 41.7 | 0.00106 | 66.7 | 0.00170 | 33.3 | 0.00106 | 83.3 | 0.00506 |
| ***Prevotella_enoeca*** | 91.7 | 0.01106 | 0.0 | 0.00000 | 16.7 | 0.00026 | 0.0 | 0.00000 | 0.0 | 0.00000 |
| ***Johnsonella_ignava*** | 91.7 | 0.00742 | 58.3 | 0.00147 | 66.7 | 0.00214 | 0.0 | 0.00000 | 8.3 | 0.00011 |
| **TM7[G-5]_sp_oral_taxon_437** | 16.7 | 0.00046 | 50.0 | 0.00503 | 25.0 | 0.00057 | 0.0 | 0.00000 | 58.3 | 0.00473 |
| ***Neisseria_sicca*** | 91.7 | 0.00558 | 41.7 | 0.00082 | 16.7 | 0.00043 | 75.0 | 0.00271 | 41.7 | 0.00108 |
| ***Prevotella_micans*** | 100.0 | 0.00734 | 0.0 | 0.00000 | 58.3 | 0.00176 | 16.7 | 0.00028 | 33.3 | 0.00115 |
| ***Streptococcus_mutans*** | 75.0 | 0.00298 | 8.3 | 0.00016 | 75.0 | 0.00602 | 16.7 | 0.00027 | 33.3 | 0.00104 |
| ***Selenomonas*_sp_oral_taxon_146** | 58.3 | 0.00798 | 0.0 | 0.00000 | 41.7 | 0.00135 | 16.7 | 0.00028 | 33.3 | 0.00073 |
| ***Treponema_lecithinolyticum*** | 16.7 | 0.00130 | 75.0 | 0.00637 | 0.0 | 0.00000 | 0.0 | 0.00000 | 33.3 | 0.00246 |
| ***Streptococcus_cristatus*** | 66.7 | 0.00258 | 33.3 | 0.00145 | 58.3 | 0.00184 | 83.3 | 0.00201 | 66.7 | 0.00192 |
| ***Selenomonas*_Genus_probe_2** | 8.3 | 0.00016 | 66.7 | 0.00558 | 8.3 | 0.00015 | 41.7 | 0.00159 | 41.7 | 0.00230 |
| ***Selenomonas*_sp_oral_taxon_138** | 58.3 | 0.00232 | 41.7 | 0.00124 | 66.7 | 0.00258 | 58.3 | 0.00148 | 58.3 | 0.00205 |
| ***Bacteroidetes*[G-5]_sp_oral_taxon_505** | 0.0 | 0.00000 | 75.0 | 0.00796 | 33.3 | 0.00139 | 0.0 | 0.00000 | 0.0 | 0.00000 |
| ***Fusobacterium_nucleatum*_subsp_animalis** | 58.3 | 0.00228 | 66.7 | 0.00319 | 50.0 | 0.00086 | 58.3 | 0.00139 | 66.7 | 0.00159 |
| ***Lactobacillus*_Genus_probe_3** | 66.7 | 0.00207 | 50.0 | 0.00172 | 75.0 | 0.00235 | 58.3 | 0.00219 | 33.3 | 0.00093 |
| ***Prevotella*_sp_oral_taxon_317** | 8.3 | 0.00044 | 33.3 | 0.00088 | 58.3 | 0.00367 | 50.0 | 0.00155 | 50.0 | 0.00268 |
| **TM7[G-1]_sp_oral_taxon_349** | 25.0 | 0.00753 | 16.7 | 0.00071 | 0.0 | 0.00000 | 0.0 | 0.00000 | 33.3 | 0.00089 |
| ***Atopobium_rimae*** | 41.7 | 0.00405 | 75.0 | 0.00278 | 8.3 | 0.00015 | 33.3 | 0.00125 | 25.0 | 0.00061 |
| ***Prevotella_oralis*** | 91.7 | 0.00632 | 58.3 | 0.00184 | 25.0 | 0.00042 | 8.3 | 0.00014 | 0.0 | 0.00000 |
| ***Leptotrichia*_sp_oral_taxon_223** | 91.7 | 0.00859 | 0.0 | 0.00000 | 0.0 | 0.00000 | 0.0 | 0.00000 | 0.0 | 0.00000 |
| ***Prevotella*_sp_oral_taxon_305** | 66.7 | 0.00224 | 41.7 | 0.00220 | 16.7 | 0.00027 | 66.7 | 0.00365 | 0.0 | 0.00000 |
| ***Veillonellaceae*[G-1]_sp_oral_taxon_155** | 50.0 | 0.00592 | 33.3 | 0.00084 | 8.3 | 0.00015 | 8.3 | 0.00016 | 33.3 | 0.00109 |
| ***Leptotrichia*_sp_oral_taxon_498** | 41.7 | 0.00154 | 25.0 | 0.00191 | 58.3 | 0.00313 | 0.0 | 0.00000 | 33.3 | 0.00158 |
| ***Prevotella*_sp_oral_taxon_300** | 75.0 | 0.00404 | 8.3 | 0.00033 | 33.3 | 0.00180 | 25.0 | 0.00054 | 50.0 | 0.00139 |
| ***Treponema*_Genus_probe_4** | 0.0 | 0.00000 | 75.0 | 0.00488 | 16.7 | 0.00025 | 0.0 | 0.00000 | 41.7 | 0.00291 |
| ***Streptococcus_anginosus*** | 41.7 | 0.00269 | 25.0 | 0.00078 | 25.0 | 0.00046 | 58.3 | 0.00251 | 41.7 | 0.00153 |
| ***Porphyromonas_catoniae*** | 58.3 | 0.00229 | 91.7 | 0.00563 | 0.0 | 0.00000 | 0.0 | 0.00000 | 0.0 | 0.00000 |
| ***Neisseria*_sp_oral_taxon_020** | 0.0 | 0.00000 | 91.7 | 0.00773 | 0.0 | 0.00000 | 0.0 | 0.00000 | 8.3 | 0.00015 |
| ***Streptococcus*_Genus_probe_2** | 66.7 | 0.00140 | 16.7 | 0.00035 | 41.7 | 0.00141 | 41.7 | 0.00097 | 58.3 | 0.00357 |
| ***Neisseria_bacilliformis*** | 75.0 | 0.00163 | 58.3 | 0.00281 | 41.7 | 0.00115 | 50.0 | 0.00193 | 0.0 | 0.00000 |
| ***Tannerella*_sp_oral_taxon_916** | 66.7 | 0.00582 | 25.0 | 0.00115 | 0.0 | 0.00000 | 8.3 | 0.00013 | 16.7 | 0.00039 |
| ***Alloprevotella*_sp_oral_taxon_912** | 0.0 | 0.00000 | 8.3 | 0.00017 | 91.7 | 0.00676 | 8.3 | 0.00016 | 16.7 | 0.00033 |
| ***Fusobacterium_nucleatum*_subsp_animalis** | 41.7 | 0.00151 | 50.0 | 0.00105 | 58.3 | 0.00170 | 25.0 | 0.00081 | 66.7 | 0.00229 |
| ***Lachnospiraceae*[G-8]_sp_oral_taxon_500** | 0.0 | 0.00000 | 8.3 | 0.00016 | 0.0 | 0.00000 | 0.0 | 0.00000 | 83.3 | 0.00713 |
| ***Selenomonas*_sp_oral_taxon_134** | 8.3 | 0.00012 | 66.7 | 0.00289 | 16.7 | 0.00027 | 41.7 | 0.00118 | 75.0 | 0.00245 |
| ***Capnocytophaga*_sp_oral_taxon_863** | 0.0 | 0.00000 | 83.3 | 0.00688 | 0.0 | 0.00000 | 0.0 | 0.00000 | 0.0 | 0.00000 |
| ***Streptococcus*_sp_oral_taxon_068** | 25.0 | 0.00092 | 25.0 | 0.00065 | 83.3 | 0.00283 | 58.3 | 0.00197 | 25.0 | 0.00045 |
| ***Treponema_maltophilum*** | 83.3 | 0.00527 | 25.0 | 0.00067 | 0.0 | 0.00000 | 0.0 | 0.00000 | 8.3 | 0.00024 |
| ***Selenomonas_dianae*** | 33.3 | 0.00376 | 41.7 | 0.00146 | 0.0 | 0.00000 | 33.3 | 0.00075 | 8.3 | 0.00017 |
| ***Kingella*_Genus_probe_2** | 58.3 | 0.00285 | 0.0 | 0.00000 | 0.0 | 0.00000 | 0.0 | 0.00000 | 75.0 | 0.00325 |
| ***Neisseria_lactamica*** | 75.0 | 0.00595 | 0.0 | 0.00000 | 0.0 | 0.00000 | 0.0 | 0.00000 | 0.0 | 0.00000 |
| ***Mitsuokella*_sp_oral_taxon_521** | 83.3 | 0.00466 | 8.3 | 0.00043 | 8.3 | 0.00010 | 8.3 | 0.00039 | 8.3 | 0.00035 |
| ***Porphyromonas*_Genus_probe_1** | 75.0 | 0.00417 | 41.7 | 0.00101 | 0.0 | 0.00000 | 8.3 | 0.00014 | 33.3 | 0.00057 |
| ***Capnocytophaga*_sp_oral_taxon_335** | 0.0 | 0.00000 | 0.0 | 0.00000 | 83.3 | 0.00555 | 8.3 | 0.00032 | 0.0 | 0.00000 |
| ***Eubacterium*[11][G-1]_infirmum** | 75.0 | 0.00378 | 50.0 | 0.00120 | 8.3 | 0.00010 | 0.0 | 0.00000 | 16.7 | 0.00052 |
| ***Tannerella*_Genus_probe** | 58.3 | 0.00287 | 41.7 | 0.00116 | 16.7 | 0.00047 | 0.0 | 0.00000 | 58.3 | 0.00106 |
| ***Prevotella_marshii*** | 91.7 | 0.00501 | 0.0 | 0.00000 | 16.7 | 0.00027 | 0.0 | 0.00000 | 8.3 | 0.00021 |
| ***Selenomonas*_Genus_probe_1** | 58.3 | 0.00485 | 25.0 | 0.00052 | 0.0 | 0.00000 | 0.0 | 0.00000 | 0.0 | 0.00000 |
| ***Erysipelothrichaceae*[G-1]_sp_oral_taxon_904** | 75.0 | 0.00504 | 8.3 | 0.00020 | 0.0 | 0.00000 | 0.0 | 0.00000 | 0.0 | 0.00000 |
| ***Bacteroidetes*[G-5]_Genus_probe** | 66.7 | 0.00436 | 8.3 | 0.00018 | 8.3 | 0.00042 | 0.0 | 0.00000 | 8.3 | 0.00028 |
| ***Anaeroglobus_geminatus*** | 33.3 | 0.00139 | 16.7 | 0.00034 | 25.0 | 0.00043 | 16.7 | 0.00026 | 41.7 | 0.00272 |
| ***Dialister*_sp_oral_taxon_502** | 0.0 | 0.00000 | 41.7 | 0.00125 | 0.0 | 0.00000 | 83.3 | 0.00373 | 0.0 | 0.00000 |
| ***Parvimonas*_sp_oral_taxon_110** | 0.0 | 0.00000 | 25.0 | 0.00057 | 58.3 | 0.00212 | 0.0 | 0.00000 | 41.7 | 0.00227 |
| ***Ottowia*_sp_oral_taxon_894** | 0.0 | 0.00000 | 66.7 | 0.00332 | 33.3 | 0.00052 | 25.0 | 0.00069 | 8.3 | 0.00035 |
| ***Actinomyces*_sp_oral_taxon_448** | 16.7 | 0.00029 | 16.7 | 0.00032 | 75.0 | 0.00335 | 33.3 | 0.00065 | 8.3 | 0.00021 |
| ***Fusobacterium*_sp_oral_taxon_205** | 50.0 | 0.00103 | 41.7 | 0.00115 | 16.7 | 0.00041 | 33.3 | 0.00054 | 75.0 | 0.00168 |
| ***Campylobacter*_Genus_probe_1** | 16.7 | 0.00257 | 25.0 | 0.00047 | 16.7 | 0.00063 | 25.0 | 0.00055 | 25.0 | 0.00047 |
| ***Tannerella*_sp_oral_taxon_916** | 50.0 | 0.00355 | 33.3 | 0.00099 | 0.0 | 0.00000 | 0.0 | 0.00000 | 8.3 | 0.00015 |
| ***Scardovia_wiggsiae*** | 66.7 | 0.00239 | 16.7 | 0.00050 | 41.7 | 0.00163 | 8.3 | 0.00013 | 0.0 | 0.00000 |
| ***Prevotella*_sp_oral_taxon_317** | 91.7 | 0.00462 | 0.0 | 0.00000 | 0.0 | 0.00000 | 0.0 | 0.00000 | 0.0 | 0.00000 |
| ***Actinomyces_massiliensis*** | 58.3 | 0.00170 | 8.3 | 0.00024 | 75.0 | 0.00268 | 0.0 | 0.00000 | 0.0 | 0.00000 |
| ***Prevotella_saccharolytica*** | 41.7 | 0.00081 | 66.7 | 0.00311 | 25.0 | 0.00066 | 0.0 | 0.00000 | 0.0 | 0.00000 |
| ***Aggregatibacter*_sp_oral_taxon_512** | 50.0 | 0.00190 | 8.3 | 0.00047 | 25.0 | 0.00095 | 33.3 | 0.00055 | 25.0 | 0.00056 |
| ***Selenomonas_flueggei*** | 33.3 | 0.00274 | 0.0 | 0.00000 | 50.0 | 0.00114 | 8.3 | 0.00013 | 16.7 | 0.00039 |
| ***Prevotella_fusca*** | 83.3 | 0.00430 | 0.0 | 0.00000 | 0.0 | 0.00000 | 0.0 | 0.00000 | 0.0 | 0.00000 |
| ***Prevotella*_sp_oral_taxon_301** | 41.7 | 0.00196 | 33.3 | 0.00174 | 8.3 | 0.00023 | 16.7 | 0.00028 | 0.0 | 0.00000 |
| ***Catonella*_sp_oral_taxon_451** | 0.0 | 0.00000 | 83.3 | 0.00374 | 0.0 | 0.00000 | 8.3 | 0.00013 | 8.3 | 0.00028 |
| ***Selenomonas_sputigena*** | 58.3 | 0.00394 | 0.0 | 0.00000 | 0.0 | 0.00000 | 0.0 | 0.00000 | 8.3 | 0.00017 |
| ***Erysipelothrichaceae*[G-1]_sp_oral_taxon_905** | 0.0 | 0.00000 | 50.0 | 0.00264 | 16.7 | 0.00039 | 0.0 | 0.00000 | 25.0 | 0.00104 |
| ***Lachnospiraceae*[G-2]_sp_oral_taxon_088** | 0.0 | 0.00000 | 0.0 | 0.00000 | 66.7 | 0.00239 | 8.3 | 0.00014 | 41.7 | 0.00152 |
| ***Bacteroidales*[G-3]_sp_oral_taxon_911** | 66.7 | 0.00403 | 0.0 | 0.00000 | 0.0 | 0.00000 | 0.0 | 0.00000 | 0.0 | 0.00000 |
| ***Atopobium_rimae*** | 33.3 | 0.00099 | 50.0 | 0.00139 | 25.0 | 0.00096 | 25.0 | 0.00048 | 8.3 | 0.00017 |
| **GN02[G-1]_sp_oral_taxon_872** | 8.3 | 0.00011 | 25.0 | 0.00051 | 50.0 | 0.00135 | 58.3 | 0.00136 | 16.7 | 0.00060 |
| ***Actinomyces*_sp_oral_taxon_171** | 33.3 | 0.00074 | 16.7 | 0.00061 | 33.3 | 0.00096 | 50.0 | 0.00160 | 0.0 | 0.00000 |
| ***Capnocytophaga*_sp_oral_taxon_332** | 58.3 | 0.00240 | 33.3 | 0.00133 | 0.0 | 0.00000 | 8.3 | 0.00015 | 0.0 | 0.00000 |
| ***Treponema*_sp_oral_taxon_262** | 0.0 | 0.00000 | 58.3 | 0.00340 | 16.7 | 0.00047 | 0.0 | 0.00000 | 0.0 | 0.00000 |
| ***Veillonellaceae*[G-1]_sp_oral_taxon_129** | 33.3 | 0.00324 | 0.0 | 0.00000 | 8.3 | 0.00012 | 8.3 | 0.00015 | 16.7 | 0.00029 |
| ***Lachnospiraceae*[G-3]_sp_oral_taxon_100** | 0.0 | 0.00000 | 66.7 | 0.00217 | 25.0 | 0.00072 | 0.0 | 0.00000 | 50.0 | 0.00088 |
| ***Bulleidia_extructa*** | 0.0 | 0.00000 | 58.3 | 0.00330 | 0.0 | 0.00000 | 0.0 | 0.00000 | 16.7 | 0.00032 |
| ***Tannerella*_sp_oral_taxon_916** | 58.3 | 0.00360 | 0.0 | 0.00000 | 0.0 | 0.00000 | 0.0 | 0.00000 | 0.0 | 0.00000 |
| ***Actinomyces*_sp_oral_taxon_170** | 41.7 | 0.00069 | 25.0 | 0.00054 | 0.0 | 0.00000 | 75.0 | 0.00235 | 0.0 | 0.00000 |
| ***Ottowia*_sp_oral_taxon_894** | 0.0 | 0.00000 | 0.0 | 0.00000 | 0.0 | 0.00000 | 91.7 | 0.00359 | 0.0 | 0.00000 |
| ***Megasphaera*_sp_oral_taxon_123** | 33.3 | 0.00348 | 0.0 | 0.00000 | 0.0 | 0.00000 | 0.0 | 0.00000 | 0.0 | 0.00000 |
| ***Peptostreptococcaceae*[11][G-7]_sp_oral_taxon_081** | 16.7 | 0.00030 | 50.0 | 0.00279 | 8.3 | 0.00016 | 0.0 | 0.00000 | 8.3 | 0.00017 |
| ***Bergeyella*_sp_oral_taxon_907** | 0.0 | 0.00000 | 66.7 | 0.00328 | 0.0 | 0.00000 | 8.3 | 0.00013 | 0.0 | 0.00000 |
| ***Porphyromonas_asaccharolytica*** | 25.0 | 0.00244 | 0.0 | 0.00000 | 8.3 | 0.00010 | 8.3 | 0.00013 | 8.3 | 0.00057 |
| ***Porphyromonas_catoniae*** | 33.3 | 0.00093 | 66.7 | 0.00230 | 0.0 | 0.00000 | 0.0 | 0.00000 | 0.0 | 0.00000 |
| ***Fretibacterium*_sp_oral_taxon_360** | 33.3 | 0.00266 | 25.0 | 0.00052 | 0.0 | 0.00000 | 0.0 | 0.00000 | 0.0 | 0.00000 |
| ***Mogibacterium_timidum*** | 50.0 | 0.00184 | 41.7 | 0.00132 | 0.0 | 0.00000 | 0.0 | 0.00000 | 0.0 | 0.00000 |
| ***Treponema_socranskii*** | 33.3 | 0.00146 | 8.3 | 0.00018 | 41.7 | 0.00148 | 0.0 | 0.00000 | 0.0 | 0.00000 |
| ***Lachnospiraceae*[G-3]_sp_oral_taxon_100** | 33.3 | 0.00088 | 41.7 | 0.00148 | 16.7 | 0.00073 | 0.0 | 0.00000 | 0.0 | 0.00000 |
| ***Treponema_vincentii*** | 58.3 | 0.00175 | 25.0 | 0.00110 | 0.0 | 0.00000 | 0.0 | 0.00000 | 0.0 | 0.00000 |
| ***Mycoplasma_faucium*** | 58.3 | 0.00267 | 8.3 | 0.00017 | 0.0 | 0.00000 | 0.0 | 0.00000 | 0.0 | 0.00000 |
| ***Veillonellaceae*_Genus_probe_3** | 25.0 | 0.00165 | 41.7 | 0.00102 | 0.0 | 0.00000 | 0.0 | 0.00000 | 8.3 | 0.00017 |
| ***Prevotella_loescheii*** | 0.0 | 0.00000 | 0.0 | 0.00000 | 50.0 | 0.00222 | 33.3 | 0.00057 | 0.0 | 0.00000 |
| ***Prevotella_loescheii*** | 0.0 | 0.00000 | 0.0 | 0.00000 | 0.0 | 0.00000 | 0.0 | 0.00000 | 50.0 | 0.00275 |
| ***Dialister*_Genus_probe_2** | 66.7 | 0.00145 | 33.3 | 0.00101 | 0.0 | 0.00000 | 16.7 | 0.00029 | 0.0 | 0.00000 |
| ***Dialister_micraerophilus*** | 0.0 | 0.00000 | 0.0 | 0.00000 | 25.0 | 0.00039 | 25.0 | 0.00116 | 50.0 | 0.00119 |
| **TM7[G-1]_sp_oral_taxon_348** | 8.3 | 0.00012 | 41.7 | 0.00227 | 0.0 | 0.00000 | 0.0 | 0.00000 | 25.0 | 0.00035 |
| ***Prevotella_dentalis*** | 0.0 | 0.00000 | 75.0 | 0.00251 | 8.3 | 0.00015 | 0.0 | 0.00000 | 0.0 | 0.00000 |
| **TM7[G-2]_sp_oral_taxon_350** | 8.3 | 0.00033 | 33.3 | 0.00206 | 0.0 | 0.00000 | 0.0 | 0.00000 | 16.7 | 0.00026 |
| ***Leptotrichia*_sp_oral_taxon_463** | 0.0 | 0.00000 | 0.0 | 0.00000 | 0.0 | 0.00000 | 0.0 | 0.00000 | 50.0 | 0.00262 |
| ***Proteus*_Genus_probe** | 16.7 | 0.00157 | 8.3 | 0.00030 | 0.0 | 0.00000 | 8.3 | 0.00013 | 16.7 | 0.00060 |
| ***Selenomonas*_sp_oral_taxon_137** | 0.0 | 0.00000 | 0.0 | 0.00000 | 66.7 | 0.00254 | 0.0 | 0.00000 | 0.0 | 0.00000 |
| ***Treponema*_sp_oral_taxon_247** | 0.0 | 0.00000 | 41.7 | 0.00240 | 0.0 | 0.00000 | 0.0 | 0.00000 | 0.0 | 0.00000 |
| **TM7[G-1]_sp_oral_taxon_348** | 16.7 | 0.00022 | 8.3 | 0.00071 | 33.3 | 0.00085 | 16.7 | 0.00034 | 16.7 | 0.00026 |
| ***Fretibacterium_fastidiosum*** | 0.0 | 0.00000 | 58.3 | 0.00212 | 0.0 | 0.00000 | 0.0 | 0.00000 | 8.3 | 0.00021 |
| ***Bacteroidetes*[G-3]_Genus_probe** | 0.0 | 0.00000 | 0.0 | 0.00000 | 8.3 | 0.00016 | 0.0 | 0.00000 | 41.7 | 0.00205 |
| ***Bacteroidetes*[G-5]_sp_oral_taxon_511** | 0.0 | 0.00000 | 0.0 | 0.00000 | 0.0 | 0.00000 | 0.0 | 0.00000 | 50.0 | 0.00217 |
| ***Mitsuokella*_sp_oral_taxon_521** | 41.7 | 0.00187 | 8.3 | 0.00015 | 0.0 | 0.00000 | 0.0 | 0.00000 | 8.3 | 0.00015 |
| ***Neisseria_flavescens*** | 16.7 | 0.00094 | 8.3 | 0.00014 | 16.7 | 0.00029 | 16.7 | 0.00028 | 25.0 | 0.00052 |
| ***Campylobacter_curvus*** | 25.0 | 0.00109 | 8.3 | 0.00018 | 0.0 | 0.00000 | 25.0 | 0.00055 | 16.7 | 0.00035 |
| ***Pseudomonas*_Genus_probe** | 8.3 | 0.00033 | 0.0 | 0.00000 | 0.0 | 0.00000 | 8.3 | 0.00014 | 16.7 | 0.00168 |
| ***Capnocytophaga*_sp_oral_taxon_902** | 8.3 | 0.00022 | 16.7 | 0.00132 | 16.7 | 0.00035 | 0.0 | 0.00000 | 8.3 | 0.00021 |
| ***Haemophilus*_Genus_probe_1** | 50.0 | 0.00200 | 0.0 | 0.00000 | 0.0 | 0.00000 | 0.0 | 0.00000 | 0.0 | 0.00000 |
| ***Actinomyces*_sp_oral_taxon_178** | 33.3 | 0.00077 | 8.3 | 0.00024 | 16.7 | 0.00072 | 8.3 | 0.00016 | 0.0 | 0.00000 |
| ***Filifactor_*Genus_probe** | 16.7 | 0.00037 | 25.0 | 0.00058 | 0.0 | 0.00000 | 0.0 | 0.00000 | 25.0 | 0.00093 |
| ***Bacteroidetes*[G-3]_sp_oral_taxon_365** | 0.0 | 0.00000 | 33.3 | 0.00186 | 0.0 | 0.00000 | 0.0 | 0.00000 | 0.0 | 0.00000 |
| ***Porphyromonas_uenonis*** | 25.0 | 0.00066 | 0.0 | 0.00000 | 0.0 | 0.00000 | 25.0 | 0.00090 | 8.3 | 0.00029 |
| ***Selenomonas_artemidis*** | 16.7 | 0.00149 | 8.3 | 0.00022 | 0.0 | 0.00000 | 8.3 | 0.00015 | 0.0 | 0.00000 |
| ***Staphylococcus*_Genus_probe_3** | 33.3 | 0.00111 | 8.3 | 0.00019 | 16.7 | 0.00040 | 8.3 | 0.00013 | 0.0 | 0.00000 |
| ***Bacteroidaceae*[G-1]_sp_oral_taxon_272** | 8.3 | 0.00122 | 0.0 | 0.00000 | 0.0 | 0.00000 | 16.7 | 0.00028 | 8.3 | 0.00032 |
| ***Fretibacterium*_Genus_probe_3** | 25.0 | 0.00063 | 41.7 | 0.00111 | 0.0 | 0.00000 | 0.0 | 0.00000 | 0.0 | 0.00000 |
| ***Treponema_amylovorum*** | 0.0 | 0.00000 | 0.0 | 0.00000 | 0.0 | 0.00000 | 58.3 | 0.00172 | 0.0 | 0.00000 |
| ***Actinomyces_meyeri*** | 0.0 | 0.00000 | 16.7 | 0.00044 | 33.3 | 0.00078 | 8.3 | 0.00013 | 8.3 | 0.00035 |
| ***Dialister*_Genus_probe_1** | 41.7 | 0.00119 | 8.3 | 0.00020 | 0.0 | 0.00000 | 0.0 | 0.00000 | 16.7 | 0.00028 |
| ***Leptotrichia*_sp_oral_taxon_215** | 16.7 | 0.00045 | 33.3 | 0.00069 | 8.3 | 0.00010 | 0.0 | 0.00000 | 16.7 | 0.00043 |
| ***Peptostreptococcaceae*[11][G-4]_sp_oral_taxon_369** | 25.0 | 0.00095 | 25.0 | 0.00056 | 0.0 | 0.00000 | 0.0 | 0.00000 | 8.3 | 0.00014 |
| ***Prevotella_buccae*** | 25.0 | 0.00052 | 41.7 | 0.00109 | 0.0 | 0.00000 | 0.0 | 0.00000 | 0.0 | 0.00000 |
| ***Actinomyces_gerencseriae*** | 25.0 | 0.00074 | 8.3 | 0.00015 | 16.7 | 0.00042 | 16.7 | 0.00028 | 0.0 | 0.00000 |
| ***Dialister*_sp_oral_taxon_119** | 33.3 | 0.00114 | 16.7 | 0.00042 | 0.0 | 0.00000 | 0.0 | 0.00000 | 0.0 | 0.00000 |
| ***Mycoplasma*_Genus_probe** | 8.3 | 0.00024 | 33.3 | 0.00092 | 0.0 | 0.00000 | 0.0 | 0.00000 | 16.7 | 0.00032 |
| ***Prevotella*_sp_oral_taxon_292** | 41.7 | 0.00144 | 0.0 | 0.00000 | 0.0 | 0.00000 | 0.0 | 0.00000 | 0.0 | 0.00000 |
| ***Lachnospiraceae*[G-5]_sp_oral_taxon_080** | 33.3 | 0.00078 | 25.0 | 0.00060 | 0.0 | 0.00000 | 0.0 | 0.00000 | 0.0 | 0.00000 |
| ***Veillonella*_sp_oral_taxon_917** | 50.0 | 0.00108 | 0.0 | 0.00000 | 8.3 | 0.00015 | 0.0 | 0.00000 | 8.3 | 0.00014 |
| **GN02[G-1]_sp_oral_taxon_871** | 16.7 | 0.00054 | 33.3 | 0.00068 | 0.0 | 0.00000 | 0.0 | 0.00000 | 8.3 | 0.00011 |
| ***Treponema_socranskii*** | 33.3 | 0.00064 | 25.0 | 0.00047 | 0.0 | 0.00000 | 0.0 | 0.00000 | 8.3 | 0.00017 |
| ***Lactobacillus*_Genus_probe_4** | 8.3 | 0.00104 | 0.0 | 0.00000 | 0.0 | 0.00000 | 8.3 | 0.00022 | 0.0 | 0.00000 |
| ***Oribacterium*_sp_oral_taxon_102** | 25.0 | 0.00048 | 8.3 | 0.00014 | 8.3 | 0.00018 | 0.0 | 0.00000 | 16.7 | 0.00046 |
| ***Bacteroides_heparinolyticus*** | 8.3 | 0.00033 | 0.0 | 0.00000 | 0.0 | 0.00000 | 8.3 | 0.00013 | 8.3 | 0.00075 |
| ***Enterococcus_faecalis*** | 25.0 | 0.00046 | 8.3 | 0.00014 | 0.0 | 0.00000 | 8.3 | 0.00027 | 8.3 | 0.00032 |
| ***Prevotella_enoeca*** | 0.0 | 0.00000 | 41.7 | 0.00119 | 0.0 | 0.00000 | 0.0 | 0.00000 | 0.0 | 0.00000 |
| ***Slackia_exigua*** | 16.7 | 0.00072 | 0.0 | 0.00000 | 0.0 | 0.00000 | 0.0 | 0.00000 | 16.7 | 0.00045 |
| ***Actinomyces*_sp_oral_taxon_848** | 25.0 | 0.00064 | 8.3 | 0.00018 | 0.0 | 0.00000 | 0.0 | 0.00000 | 16.7 | 0.00032 |
| ***Prevotella_intermedia*** | 16.7 | 0.00050 | 16.7 | 0.00061 | 0.0 | 0.00000 | 0.0 | 0.00000 | 0.0 | 0.00000 |
| ***Clostridiales*[F-2][G-3]_sp_oral_taxon_381** | 8.3 | 0.00044 | 0.0 | 0.00000 | 0.0 | 0.00000 | 8.3 | 0.00014 | 25.0 | 0.00046 |
| ***Actinomyces*_sp_oral_taxon_175** | 0.0 | 0.00000 | 0.0 | 0.00000 | 33.3 | 0.00092 | 0.0 | 0.00000 | 8.3 | 0.00011 |
| ***Prevotella_marshii*** | 0.0 | 0.00000 | 25.0 | 0.00103 | 0.0 | 0.00000 | 0.0 | 0.00000 | 0.0 | 0.00000 |
| ***Finegoldia_magna*** | 16.7 | 0.00049 | 8.3 | 0.00017 | 0.0 | 0.00000 | 0.0 | 0.00000 | 8.3 | 0.00029 |
| ***Eggerthia_catenaformis*** | 0.0 | 0.00000 | 16.7 | 0.00093 | 0.0 | 0.00000 | 0.0 | 0.00000 | 0.0 | 0.00000 |
| ***Actinomyces_naeslundii*** | 0.0 | 0.00000 | 0.0 | 0.00000 | 41.7 | 0.00061 | 0.0 | 0.00000 | 8.3 | 0.00032 |
| ***Alloprevotella*_sp_oral_taxon_913** | 0.0 | 0.00000 | 0.0 | 0.00000 | 50.0 | 0.00092 | 0.0 | 0.00000 | 0.0 | 0.00000 |
| ***Capnocytophaga*_sp_oral_taxon_864** | 0.0 | 0.00000 | 33.3 | 0.00081 | 0.0 | 0.00000 | 0.0 | 0.00000 | 8.3 | 0.00011 |
| ***Lachnospiraceae*[G-7]_sp_oral_taxon_163** | 33.3 | 0.00070 | 8.3 | 0.00015 | 0.0 | 0.00000 | 0.0 | 0.00000 | 0.0 | 0.00000 |
| ***Treponema_socranskii*** | 0.0 | 0.00000 | 0.0 | 0.00000 | 8.3 | 0.00010 | 0.0 | 0.00000 | 33.3 | 0.00075 |
| ***Actinomyces*_sp_oral_taxon_896** | 0.0 | 0.00000 | 0.0 | 0.00000 | 25.0 | 0.00085 | 0.0 | 0.00000 | 0.0 | 0.00000 |
| ***Capnocytophaga*_sp_oral_taxon_336** | 0.0 | 0.00000 | 25.0 | 0.00085 | 0.0 | 0.00000 | 0.0 | 0.00000 | 0.0 | 0.00000 |
| ***Eubacterium*[11][G-6]_nodatum** | 8.3 | 0.00033 | 8.3 | 0.00015 | 8.3 | 0.00036 | 0.0 | 0.00000 | 0.0 | 0.00000 |
| ***Prevotella*_sp_oral_taxon_306** | 8.3 | 0.00016 | 8.3 | 0.00024 | 8.3 | 0.00012 | 16.7 | 0.00030 | 0.0 | 0.00000 |
| ***Prevotella*_sp_oral_taxon_443** | 0.0 | 0.00000 | 16.7 | 0.00051 | 8.3 | 0.00015 | 0.0 | 0.00000 | 8.3 | 0.00014 |
| ***Bacteroidetes*[G-3]_sp_oral_taxon_503** | 0.0 | 0.00000 | 0.0 | 0.00000 | 16.7 | 0.00076 | 0.0 | 0.00000 | 0.0 | 0.00000 |
| ***Clostridiales*[F-2][G-1]_sp_oral_taxon_075** | 8.3 | 0.00044 | 8.3 | 0.00018 | 0.0 | 0.00000 | 0.0 | 0.00000 | 8.3 | 0.00014 |
| ***Porphyromonas*_Genus_probe_3** | 0.0 | 0.00000 | 0.0 | 0.00000 | 0.0 | 0.00000 | 25.0 | 0.00074 | 0.0 | 0.00000 |
| ***Treponema*_Genus_probe_5** | 8.3 | 0.00022 | 16.7 | 0.00050 | 0.0 | 0.00000 | 0.0 | 0.00000 | 0.0 | 0.00000 |
| ***Olsenella*_sp_oral_taxon_807** | 8.3 | 0.00022 | 16.7 | 0.00029 | 8.3 | 0.00020 | 0.0 | 0.00000 | 0.0 | 0.00000 |
| ***Megasphaera*_sp_oral_taxon_123** | 0.0 | 0.00000 | 25.0 | 0.00070 | 0.0 | 0.00000 | 0.0 | 0.00000 | 0.0 | 0.00000 |
| ***Treponema*_Genus_probe_1** | 8.3 | 0.00013 | 16.7 | 0.00039 | 0.0 | 0.00000 | 0.0 | 0.00000 | 8.3 | 0.00014 |
| ***Cryptobacterium_curtum*** | 16.7 | 0.00039 | 0.0 | 0.00000 | 8.3 | 0.00010 | 0.0 | 0.00000 | 8.3 | 0.00017 |
| ***Treponema*_sp_oral_taxon_268** | 0.0 | 0.00000 | 8.3 | 0.00030 | 16.7 | 0.00036 | 0.0 | 0.00000 | 0.0 | 0.00000 |
| ***Actinomyces_viscosus*** | 0.0 | 0.00000 | 8.3 | 0.00031 | 8.3 | 0.00010 | 8.3 | 0.00013 | 8.3 | 0.00011 |
| ***Actinomyces_georgiae*** | 33.3 | 0.00062 | 0.0 | 0.00000 | 0.0 | 0.00000 | 0.0 | 0.00000 | 0.0 | 0.00000 |
| ***Johnsonella*_Genus_probe** | 16.7 | 0.00029 | 0.0 | 0.00000 | 16.7 | 0.00031 | 0.0 | 0.00000 | 0.0 | 0.00000 |
| ***Treponema*_Genus_probe_3** | 33.3 | 0.00060 | 0.0 | 0.00000 | 0.0 | 0.00000 | 0.0 | 0.00000 | 0.0 | 0.00000 |
| ***Capnocytophaga*_sp_oral_taxon_903** | 0.0 | 0.00000 | 8.3 | 0.00015 | 0.0 | 0.00000 | 25.0 | 0.00044 | 0.0 | 0.00000 |
| ***Actinomyces_israelii*** | 25.0 | 0.00038 | 0.0 | 0.00000 | 8.3 | 0.00010 | 0.0 | 0.00000 | 8.3 | 0.00011 |
| ***Peptococcus*_sp_oral_taxon_167** | 0.0 | 0.00000 | 0.0 | 0.00000 | 16.7 | 0.00027 | 0.0 | 0.00000 | 16.7 | 0.00032 |
| ***Treponema*_sp_oral_taxon_270** | 0.0 | 0.00000 | 25.0 | 0.00058 | 0.0 | 0.00000 | 0.0 | 0.00000 | 0.0 | 0.00000 |
| ***Veillonellaceae*_Genus_probe_2** | 16.7 | 0.00057 | 0.0 | 0.00000 | 0.0 | 0.00000 | 0.0 | 0.00000 | 0.0 | 0.00000 |
| ***Prevotella_*sp_oral_taxon_475** | 0.0 | 0.00000 | 0.0 | 0.00000 | 0.0 | 0.00000 | 16.7 | 0.00055 | 0.0 | 0.00000 |
| ***Stenotrophomonas_maltophilia*** | 8.3 | 0.00019 | 0.0 | 0.00000 | 0.0 | 0.00000 | 0.0 | 0.00000 | 8.3 | 0.00032 |
| ***Lachnoanaerobaculum*_sp_oral_taxon_089** | 0.0 | 0.00000 | 8.3 | 0.00048 | 0.0 | 0.00000 | 0.0 | 0.00000 | 0.0 | 0.00000 |
| ***Streptococcus_*sp_oral_taxon_069** | 8.3 | 0.00032 | 0.0 | 0.00000 | 0.0 | 0.00000 | 0.0 | 0.00000 | 8.3 | 0.00014 |
| ***Arcanobacterium_haemolyticum*** | 0.0 | 0.00000 | 0.0 | 0.00000 | 8.3 | 0.00016 | 8.3 | 0.00029 | 0.0 | 0.00000 |
| ***Lactobacillus*_Genus_probe_5** | 0.0 | 0.00000 | 8.3 | 0.00046 | 0.0 | 0.00000 | 0.0 | 0.00000 | 0.0 | 0.00000 |
| ***Peptostreptococcaceae*[13][G-1]_sp_oral_taxon_113** | 8.3 | 0.00026 | 0.0 | 0.00000 | 0.0 | 0.00000 | 8.3 | 0.00020 | 0.0 | 0.00000 |
| ***Eubacterium*[11][G-7]_yurii** | 8.3 | 0.00011 | 0.0 | 0.00000 | 0.0 | 0.00000 | 0.0 | 0.00000 | 16.7 | 0.00033 |
| ***Selenomonas_sputigena*** | 0.0 | 0.00000 | 8.3 | 0.00015 | 0.0 | 0.00000 | 16.7 | 0.00027 | 0.0 | 0.00000 |
| ***Selenomonas_sputigena*** | 0.0 | 0.00000 | 0.0 | 0.00000 | 0.0 | 0.00000 | 16.7 | 0.00042 | 0.0 | 0.00000 |
| ***Treponema*_sp_oral_taxon_236** | 0.0 | 0.00000 | 16.7 | 0.00042 | 0.0 | 0.00000 | 0.0 | 0.00000 | 0.0 | 0.00000 |
| ***Veillonellaceae*[G-1]_sp_oral_taxon_483** | 8.3 | 0.00011 | 0.0 | 0.00000 | 8.3 | 0.00030 | 0.0 | 0.00000 | 0.0 | 0.00000 |
| ***Porphyromonas_gingivalis*** | 16.7 | 0.00039 | 0.0 | 0.00000 | 0.0 | 0.00000 | 0.0 | 0.00000 | 0.0 | 0.00000 |
| ***Atopobium*_sp_oral_taxon_199** | 8.3 | 0.00022 | 0.0 | 0.00000 | 8.3 | 0.00015 | 0.0 | 0.00000 | 0.0 | 0.00000 |
| ***Tannerella*_sp_oral_taxon_916** | 0.0 | 0.00000 | 8.3 | 0.00035 | 0.0 | 0.00000 | 0.0 | 0.00000 | 0.0 | 0.00000 |
| ***Eikenella*_sp_oral_taxon_011** | 0.0 | 0.00000 | 0.0 | 0.00000 | 16.7 | 0.00035 | 0.0 | 0.00000 | 0.0 | 0.00000 |
| **TM7[G-4]_sp_oral_taxon_355** | 0.0 | 0.00000 | 0.0 | 0.00000 | 8.3 | 0.00014 | 0.0 | 0.00000 | 8.3 | 0.00021 |
| ***Peptostreptococcaceae*[11][G-2]_sp_oral_taxon_091** | 0.0 | 0.00000 | 16.7 | 0.00034 | 0.0 | 0.00000 | 0.0 | 0.00000 | 0.0 | 0.00000 |
| ***Peptostreptococcaceae*[11][G-3]_sp_oral_taxon_495** | 0.0 | 0.00000 | 8.3 | 0.00033 | 0.0 | 0.00000 | 0.0 | 0.00000 | 0.0 | 0.00000 |
| ***Prevotella*_sp_oral_taxon_317** | 0.0 | 0.00000 | 0.0 | 0.00000 | 0.0 | 0.00000 | 16.7 | 0.00033 | 0.0 | 0.00000 |
| ***Proteus_mirabilis*** | 8.3 | 0.00022 | 0.0 | 0.00000 | 0.0 | 0.00000 | 0.0 | 0.00000 | 8.3 | 0.00011 |
| ***Peptoniphilus*_sp_oral_taxon_836** | 0.0 | 0.00000 | 0.0 | 0.00000 | 0.0 | 0.00000 | 8.3 | 0.00032 | 0.0 | 0.00000 |
| ***Fusobacterium*_Genus_probe_1** | 0.0 | 0.00000 | 8.3 | 0.00017 | 0.0 | 0.00000 | 0.0 | 0.00000 | 8.3 | 0.00015 |
| ***Prevotella_multiformis*** | 0.0 | 0.00000 | 8.3 | 0.00018 | 0.0 | 0.00000 | 0.0 | 0.00000 | 8.3 | 0.00014 |
| ***Lachnospiraceae*[G-3]_sp_oral_taxon_100** | 0.0 | 0.00000 | 0.0 | 0.00000 | 16.7 | 0.00032 | 0.0 | 0.00000 | 0.0 | 0.00000 |
| ***Porphyromonas_catoniae*** | 0.0 | 0.00000 | 8.3 | 0.00031 | 0.0 | 0.00000 | 0.0 | 0.00000 | 0.0 | 0.00000 |
| ***Peptoniphilus*_Genus_probe** | 16.7 | 0.00029 | 0.0 | 0.00000 | 0.0 | 0.00000 | 0.0 | 0.00000 | 0.0 | 0.00000 |
| ***Veillonellaceae*[G-1]_sp_oral_taxon_135** | 8.3 | 0.00011 | 0.0 | 0.00000 | 0.0 | 0.00000 | 0.0 | 0.00000 | 8.3 | 0.00015 |
| ***Prevotella*_sp_oral_taxon_300** | 0.0 | 0.00000 | 0.0 | 0.00000 | 16.7 | 0.00025 | 0.0 | 0.00000 | 0.0 | 0.00000 |
| ***Bacteroidetes*[G-3]_sp_oral_taxon_280** | 0.0 | 0.00000 | 8.3 | 0.00022 | 0.0 | 0.00000 | 0.0 | 0.00000 | 0.0 | 0.00000 |
| ***Peptoniphilus_asaccharolyticus*** | 0.0 | 0.00000 | 0.0 | 0.00000 | 0.0 | 0.00000 | 0.0 | 0.00000 | 8.3 | 0.00021 |
| ***Treponema*_sp_oral_taxon_226** | 0.0 | 0.00000 | 0.0 | 0.00000 | 0.0 | 0.00000 | 0.0 | 0.00000 | 8.3 | 0.00021 |
| ***Stomatobaculum*_sp_oral_taxon_373** | 0.0 | 0.00000 | 0.0 | 0.00000 | 8.3 | 0.00021 | 0.0 | 0.00000 | 0.0 | 0.00000 |
| ***Bacteroidetes*[G-5]_sp_oral_taxon_511** | 0.0 | 0.00000 | 0.0 | 0.00000 | 8.3 | 0.00021 | 0.0 | 0.00000 | 0.0 | 0.00000 |
| ***Dolosigranulum_pigrum*** | 8.3 | 0.00019 | 0.0 | 0.00000 | 0.0 | 0.00000 | 0.0 | 0.00000 | 0.0 | 0.00000 |
| ***Lactobacillus_coleohominis*** | 0.0 | 0.00000 | 0.0 | 0.00000 | 8.3 | 0.00018 | 0.0 | 0.00000 | 0.0 | 0.00000 |
| ***Streptococcus*_sp_oral_taxon_487** | 0.0 | 0.00000 | 0.0 | 0.00000 | 8.3 | 0.00018 | 0.0 | 0.00000 | 0.0 | 0.00000 |
| ***Aggregatibacter_actinomycetemcomitans*** | 0.0 | 0.00000 | 0.0 | 0.00000 | 0.0 | 0.00000 | 0.0 | 0.00000 | 8.3 | 0.00017 |
| ***Helicobacter*_Genus_probe** | 0.0 | 0.00000 | 0.0 | 0.00000 | 0.0 | 0.00000 | 0.0 | 0.00000 | 8.3 | 0.00017 |
| ***Porphyromonas*_sp_oral_taxon_277** | 0.0 | 0.00000 | 8.3 | 0.00017 | 0.0 | 0.00000 | 0.0 | 0.00000 | 0.0 | 0.00000 |
| ***Veillonellaceae*[G-1]_sp_oral_taxon_155** | 0.0 | 0.00000 | 0.0 | 0.00000 | 0.0 | 0.00000 | 0.0 | 0.00000 | 8.3 | 0.00017 |
| ***Dialister*_sp_oral_taxon_502** | 0.0 | 0.00000 | 8.3 | 0.00017 | 0.0 | 0.00000 | 0.0 | 0.00000 | 0.0 | 0.00000 |
| ***Lactobacillus*_Genus_probe_1** | 8.3 | 0.00017 | 0.0 | 0.00000 | 0.0 | 0.00000 | 0.0 | 0.00000 | 0.0 | 0.00000 |
| ***Prevotella*_sp_oral_taxon_296** | 8.3 | 0.00017 | 0.0 | 0.00000 | 0.0 | 0.00000 | 0.0 | 0.00000 | 0.0 | 0.00000 |
| ***Pyramidobacter_piscolens*** | 8.3 | 0.00016 | 0.0 | 0.00000 | 0.0 | 0.00000 | 0.0 | 0.00000 | 0.0 | 0.00000 |
| ***Actinomyces*_sp_oral_taxon_414** | 0.0 | 0.00000 | 8.3 | 0.00015 | 0.0 | 0.00000 | 0.0 | 0.00000 | 0.0 | 0.00000 |
| ***Actinomyces*_sp_oral_taxon_897** | 0.0 | 0.00000 | 8.3 | 0.00015 | 0.0 | 0.00000 | 0.0 | 0.00000 | 0.0 | 0.00000 |
| ***Selenomonas*_sp_oral_taxon_478** | 0.0 | 0.00000 | 8.3 | 0.00015 | 0.0 | 0.00000 | 0.0 | 0.00000 | 0.0 | 0.00000 |
| ***Capnocytophaga*_sp_oral_taxon_380** | 0.0 | 0.00000 | 0.0 | 0.00000 | 8.3 | 0.00015 | 0.0 | 0.00000 | 0.0 | 0.00000 |
| ***Lactobacillus_salivarius*** | 0.0 | 0.00000 | 0.0 | 0.00000 | 0.0 | 0.00000 | 0.0 | 0.00000 | 8.3 | 0.00015 |
| ***Lactobacillus_brevis*** | 0.0 | 0.00000 | 0.0 | 0.00000 | 0.0 | 0.00000 | 8.3 | 0.00015 | 0.0 | 0.00000 |
| ***Bifidobacterium*_animalis_subsp_lactis** | 0.0 | 0.00000 | 0.0 | 0.00000 | 0.0 | 0.00000 | 8.3 | 0.00014 | 0.0 | 0.00000 |
| ***Bifidobacterium_longum*** | 0.0 | 0.00000 | 0.0 | 0.00000 | 0.0 | 0.00000 | 8.3 | 0.00014 | 0.0 | 0.00000 |
| ***Capnocytophaga*_Genus_probe_1** | 0.0 | 0.00000 | 0.0 | 0.00000 | 0.0 | 0.00000 | 8.3 | 0.00014 | 0.0 | 0.00000 |
| ***Sneathia_amnionii*** | 0.0 | 0.00000 | 8.3 | 0.00014 | 0.0 | 0.00000 | 0.0 | 0.00000 | 0.0 | 0.00000 |
| ***Actinomyces*_Genus_probe_2** | 0.0 | 0.00000 | 0.0 | 0.00000 | 0.0 | 0.00000 | 8.3 | 0.00013 | 0.0 | 0.00000 |
| ***Lactobacillus_vaginalis*** | 0.0 | 0.00000 | 0.0 | 0.00000 | 0.0 | 0.00000 | 8.3 | 0.00013 | 0.0 | 0.00000 |
| ***Aggregatibacter_paraphrophilus*** | 8.3 | 0.00013 | 0.0 | 0.00000 | 0.0 | 0.00000 | 0.0 | 0.00000 | 0.0 | 0.00000 |
| ***Prevotella*_sp_oral_taxon_304** | 8.3 | 0.00013 | 0.0 | 0.00000 | 0.0 | 0.00000 | 0.0 | 0.00000 | 0.0 | 0.00000 |
| ***Lactobacillus*_Genus_probe_2** | 8.3 | 0.00012 | 0.0 | 0.00000 | 0.0 | 0.00000 | 0.0 | 0.00000 | 0.0 | 0.00000 |
| ***Treponema_denticola*** | 0.0 | 0.00000 | 0.0 | 0.00000 | 8.3 | 0.00012 | 0.0 | 0.00000 | 0.0 | 0.00000 |
| ***Bifidobacterium_dentium*** | 8.3 | 0.00011 | 0.0 | 0.00000 | 0.0 | 0.00000 | 0.0 | 0.00000 | 0.0 | 0.00000 |
| ***Veillonellaceae*_Genus_probe_1** | 8.3 | 0.00011 | 0.0 | 0.00000 | 0.0 | 0.00000 | 0.0 | 0.00000 | 0.0 | 0.00000 |
| ***Selenomonas_sputigena*** | 8.3 | 0.00011 | 0.0 | 0.00000 | 0.0 | 0.00000 | 0.0 | 0.00000 | 0.0 | 0.00000 |
| **TM7[G-1]_sp_oral_taxon_488** | 8.3 | 0.00011 | 0.0 | 0.00000 | 0.0 | 0.00000 | 0.0 | 0.00000 | 0.0 | 0.00000 |
| ***Fretibacterium*_Genus_probe_2** | 0.0 | 0.00000 | 0.0 | 0.00000 | 0.0 | 0.00000 | 0.0 | 0.00000 | 8.3 | 0.00011 |
| ***Clostridiales*[F-2][G-3]_sp_oral_taxon_366** | 0.0 | 0.00000 | 0.0 | 0.00000 | 0.0 | 0.00000 | 0.0 | 0.00000 | 8.3 | 0.00011 |
| ***Eubacterium*[11][G-5]_saphenum** | 0.0 | 0.00000 | 0.0 | 0.00000 | 0.0 | 0.00000 | 0.0 | 0.00000 | 8.3 | 0.00011 |
| ***Olsenella*_Genus_probe** | 0.0 | 0.00000 | 0.0 | 0.00000 | 0.0 | 0.00000 | 0.0 | 0.00000 | 8.3 | 0.00011 |
| ***Staphylococcus*_Genus_probe_1** | 0.0 | 0.00000 | 0.0 | 0.00000 | 8.3 | 0.00010 | 0.0 | 0.00000 | 0.0 | 0.00000 |
| ***Olsenella*_sp_oral_taxon_809** | 0.0 | 0.00000 | 0.0 | 0.00000 | 8.3 | 0.00010 | 0.0 | 0.00000 | 0.0 | 0.00000 |
